# Supplementary material for: Examining how goals of care communication are conducted between doctors and patients with severe acute illness in hospital settings: A realist systematic review
Source: PLoS One. 2024 Mar 18;19(3):e0299933. doi: 10.1371/journal.pone.0299933 (PMC10947705; doi:10.1371/journal.pone.0299933)
Supplement: S1 Appendix — (DOCX) [file pone.0299933.s003.docx]

**Appendix 1: Literature Search Strategy (MEDLINE)**

| **Searches** | **Results** |  |
| --- | --- | --- |
| 1 | exp Life Support Care/ |  |
| 2 | life support care.mp. [mp=title, book title, abstract, original title, name of substance word, subject heading word, floating sub-heading word, keyword heading word, organism supplementary concept word, protocol supplementary concept word, rare disease supplementary concept word, unique identifier, synonyms] |  |
| 3 | "life sustaining".mp. |  |
| 4 | "treatment escalation".mp. |  |
| 5 | "ceiling of treatment".mp. |  |
| 6 | "ceiling of care".mp. [mp=title, book title, abstract, original title, name of substance word, subject heading word, floating sub-heading word, keyword heading word, organism supplementary concept word, protocol supplementary concept word, rare disease supplementary concept word, unique identifier, synonyms] |  |
| 7 | "treatment limitation".mp. [mp=title, book title, abstract, original title, name of substance word, subject heading word, floating sub-heading word, keyword heading word, organism supplementary concept word, protocol supplementary concept word, rare disease supplementary concept word, unique identifier, synonyms] |  |
| 8 | "goals of treatment".mp. |  |
| 9 | "goals of care".mp. |  |
| 10 | treatment priorit*.mp. |  |
| 11 | exp Patient Care Planning/ |  |
| 12 | patient care planning.mp. [mp=title, book title, abstract, original title, name of substance word, subject heading word, floating sub-heading word, keyword heading word, organism supplementary concept word, protocol supplementary concept word, rare disease supplementary concept word, unique identifier, synonyms] |  |
| 13 | "shared understanding".mp. |  |
| 14 | exp Physician-Patient Relations/ |  |
| 15 | physician-patient relations.mp. [mp=title, book title, abstract, original title, name of substance word, subject heading word, floating sub-heading word, keyword heading word, organism supplementary concept word, protocol supplementary concept word, rare disease supplementary concept word, unique identifier, synonyms] |  |
| 16 | exp Professional-Patient Relations/ |  |
| 17 | professional-patient relations.mp. [mp=title, book title, abstract, original title, name of substance word, subject heading word, floating sub-heading word, keyword heading word, organism supplementary concept word, protocol supplementary concept word, rare disease supplementary concept word, unique identifier, synonyms] |  |
| 18 | exp Decision Making, Shared/ |  |
| 19 | "shared decision making".mp. [mp=title, book title, abstract, original title, name of substance word, subject heading word, floating sub-heading word, keyword heading word, organism supplementary concept word, protocol supplementary concept word, rare disease supplementary concept word, unique identifier, synonyms] |  |
| 20 | information provision.mp. |  |
| 21 | exp Patient Education as Topic/ |  |
| 22 | "patient education".mp. [mp=title, book title, abstract, original title, name of substance word, subject heading word, floating sub-heading word, keyword heading word, organism supplementary concept word, protocol supplementary concept word, rare disease supplementary concept word, unique identifier, synonyms] |  |
| 23 | exp Information Literacy/ |  |
| 24 | information literacy.mp. [mp=title, book title, abstract, original title, name of substance word, subject heading word, floating sub-heading word, keyword heading word, organism supplementary concept word, protocol supplementary concept word, rare disease supplementary concept word, unique identifier, synonyms] |  |
| 25 | exp Motivation/ |  |
| 26 | motivation.mp. [mp=title, book title, abstract, original title, name of substance word, subject heading word, floating sub-heading word, keyword heading word, organism supplementary concept word, protocol supplementary concept word, rare disease supplementary concept word, unique identifier, synonyms] |  |
| 27 | exp Empowerment/ |  |
| 28 | empower*.mp. [mp=title, book title, abstract, original title, name of substance word, subject heading word, floating sub-heading word, keyword heading word, organism supplementary concept word, protocol supplementary concept word, rare disease supplementary concept word, unique identifier, synonyms] |  |
| 29 | exp Health Literacy/ |  |
| 30 | health literacy.mp. [mp=title, book title, abstract, original title, name of substance word, subject heading word, floating sub-heading word, keyword heading word, organism supplementary concept word, protocol supplementary concept word, rare disease supplementary concept word, unique identifier, synonyms] |  |
| 31 | personal*.mp. |  |
| 32 | exp "Patient Acceptance of Health Care"/ |  |
| 33 | exp Bias/ |  |
| 34 | bias.mp. [mp=title, book title, abstract, original title, name of substance word, subject heading word, floating sub-heading word, keyword heading word, organism supplementary concept word, protocol supplementary concept word, rare disease supplementary concept word, unique identifier, synonyms] |  |
| 35 | exp Judgment/ |  |
| 36 | judgment.mp. [mp=title, book title, abstract, original title, name of substance word, subject heading word, floating sub-heading word, keyword heading word, organism supplementary concept word, protocol supplementary concept word, rare disease supplementary concept word, unique identifier, synonyms] |  |
| 37 | exp Family Conflict/ |  |
| 38 | family conflict.mp. [mp=title, book title, abstract, original title, name of substance word, subject heading word, floating sub-heading word, keyword heading word, organism supplementary concept word, protocol supplementary concept word, rare disease supplementary concept word, unique identifier, synonyms] |  |
| 39 | influenc*.mp. |  |
| 40 | exp Prejudice/ |  |
| 41 | prejudice.mp. [mp=title, book title, abstract, original title, name of substance word, subject heading word, floating sub-heading word, keyword heading word, organism supplementary concept word, protocol supplementary concept word, rare disease supplementary concept word, unique identifier, synonyms] |  |
| 42 | exp Trust/ |  |
| 43 | trust.mp. [mp=title, book title, abstract, original title, name of substance word, subject heading word, floating sub-heading word, keyword heading word, organism supplementary concept word, protocol supplementary concept word, rare disease supplementary concept word, unique identifier, synonyms] |  |
| 44 | experience.mp. |  |
| 45 | confidence.mp. |  |
| 46 | exp Communication/ or communication skills.mp. |  |
| 47 | exp Education, Medical/ |  |
| 48 | "medical education".mp. [mp=title, book title, abstract, original title, name of substance word, subject heading word, floating sub-heading word, keyword heading word, organism supplementary concept word, protocol supplementary concept word, rare disease supplementary concept word, unique identifier, synonyms] |  |
| 49 | exp Self Concept/ |  |
| 50 | self concept.mp. [mp=title, book title, abstract, original title, name of substance word, subject heading word, floating sub-heading word, keyword heading word, organism supplementary concept word, protocol supplementary concept word, rare disease supplementary concept word, unique identifier, synonyms] |  |
| 51 | exp Social Skills/ |  |
| 52 | social skills.mp. [mp=title, book title, abstract, original title, name of substance word, subject heading word, floating sub-heading word, keyword heading word, organism supplementary concept word, protocol supplementary concept word, rare disease supplementary concept word, unique identifier, synonyms] |  |
| 53 | exp Frailty/ |  |
| 54 | frail*.mp. [mp=title, book title, abstract, original title, name of substance word, subject heading word, floating sub-heading word, keyword heading word, organism supplementary concept word, protocol supplementary concept word, rare disease supplementary concept word, unique identifier, synonyms] |  |
| 55 | exp Frail Elderly/ |  |
| 56 | exp Comorbidity/ |  |
| 57 | Comorb*.mp. [mp=title, book title, abstract, original title, name of substance word, subject heading word, floating sub-heading word, keyword heading word, organism supplementary concept word, protocol supplementary concept word, rare disease supplementary concept word, unique identifier, synonyms] |  |
| 58 | exp Uncertainty/ |  |
| 59 | uncertain*.mp. [mp=title, book title, abstract, original title, name of substance word, subject heading word, floating sub-heading word, keyword heading word, organism supplementary concept word, protocol supplementary concept word, rare disease supplementary concept word, unique identifier, synonyms] |  |
| 60 | "end of life".mp. |  |
| 61 | "treatment burden".mp. |  |
| 62 | exp "Attitude of Health Personnel"/ |  |
| 63 | attitude.mp. [mp=title, book title, abstract, original title, name of substance word, subject heading word, floating sub-heading word, keyword heading word, organism supplementary concept word, protocol supplementary concept word, rare disease supplementary concept word, unique identifier, synonyms] |  |
| 64 | exp Patient Participation/ |  |
| 65 | patient particip*.mp. [mp=title, book title, abstract, original title, name of substance word, subject heading word, floating sub-heading word, keyword heading word, organism supplementary concept word, protocol supplementary concept word, rare disease supplementary concept word, unique identifier, synonyms] |  |
| 66 | exp Personal Autonomy/ |  |
| 67 | personal autonomy.mp. [mp=title, book title, abstract, original title, name of substance word, subject heading word, floating sub-heading word, keyword heading word, organism supplementary concept word, protocol supplementary concept word, rare disease supplementary concept word, unique identifier, synonyms] |  |
| 68 | exp Patient-Centered Care/ |  |
| 69 | "patient cent*".mp. [mp=title, book title, abstract, original title, name of substance word, subject heading word, floating sub-heading word, keyword heading word, organism supplementary concept word, protocol supplementary concept word, rare disease supplementary concept word, unique identifier, synonyms] |  |
| 70 | incentiv*.mp. |  |
| 71 | exp Organizational Culture/ |  |
| 72 | "organi?ational culture".mp. [mp=title, book title, abstract, original title, name of substance word, subject heading word, floating sub-heading word, keyword heading word, organism supplementary concept word, protocol supplementary concept word, rare disease supplementary concept word, unique identifier, synonyms] |  |
| 73 | funding.mp. |  |
| 74 | invest*.mp. |  |
| 75 | "organi?ational support".mp. |  |
| 76 | exp Respiration, Artificial/ |  |
| 77 | intubation.mp. [mp=title, book title, abstract, original title, name of substance word, subject heading word, floating sub-heading word, keyword heading word, organism supplementary concept word, protocol supplementary concept word, rare disease supplementary concept word, unique identifier, synonyms] |  |
| 78 | "mechanical ventilation".mp. [mp=title, book title, abstract, original title, name of substance word, subject heading word, floating sub-heading word, keyword heading word, organism supplementary concept word, protocol supplementary concept word, rare disease supplementary concept word, unique identifier, synonyms] |  |
| 79 | exp Intensive Care Units/ |  |
| 80 | intensive care unit*.mp. [mp=title, book title, abstract, original title, name of substance word, subject heading word, floating sub-heading word, keyword heading word, organism supplementary concept word, protocol supplementary concept word, rare disease supplementary concept word, unique identifier, synonyms] |  |
| 81 | ICU.mp. [mp=title, book title, abstract, original title, name of substance word, subject heading word, floating sub-heading word, keyword heading word, organism supplementary concept word, protocol supplementary concept word, rare disease supplementary concept word, unique identifier, synonyms] |  |
| 82 | "organ support".mp. [mp=title, book title, abstract, original title, name of substance word, subject heading word, floating sub-heading word, keyword heading word, organism supplementary concept word, protocol supplementary concept word, rare disease supplementary concept word, unique identifier, synonyms] |  |
| 83 | exp Critical Illness/ |  |
| 84 | critical illness.mp. [mp=title, book title, abstract, original title, name of substance word, subject heading word, floating sub-heading word, keyword heading word, organism supplementary concept word, protocol supplementary concept word, rare disease supplementary concept word, unique identifier, synonyms] |  |
| 85 | exp Critical Care/ |  |
| 86 | critical care.mp. [mp=title, book title, abstract, original title, name of substance word, subject heading word, floating sub-heading word, keyword heading word, organism supplementary concept word, protocol supplementary concept word, rare disease supplementary concept word, unique identifier, synonyms] |  |
| 87 | exp Cardiopulmonary Resuscitation/ |  |
| 88 | "cardiopulmonary resuscitation".mp. [mp=title, book title, abstract, original title, name of substance word, subject heading word, floating sub-heading word, keyword heading word, organism supplementary concept word, protocol supplementary concept word, rare disease supplementary concept word, unique identifier, synonyms] |  |
| 89 | "do not attempt cardiopulmonary resuscitation".mp. [mp=title, book title, abstract, original title, name of substance word, subject heading word, floating sub-heading word, keyword heading word, organism supplementary concept word, protocol supplementary concept word, rare disease supplementary concept word, unique identifier, synonyms] |  |
| 90 | DNR.mp. [mp=title, book title, abstract, original title, name of substance word, subject heading word, floating sub-heading word, keyword heading word, organism supplementary concept word, protocol supplementary concept word, rare disease supplementary concept word, unique identifier, synonyms] |  |
| 91 | DNAR.mp. [mp=title, book title, abstract, original title, name of substance word, subject heading word, floating sub-heading word, keyword heading word, organism supplementary concept word, protocol supplementary concept word, rare disease supplementary concept word, unique identifier, synonyms] |  |
| 92 | DNACPR.mp. [mp=title, book title, abstract, original title, name of substance word, subject heading word, floating sub-heading word, keyword heading word, organism supplementary concept word, protocol supplementary concept word, rare disease supplementary concept word, unique identifier, synonyms] |  |
| 93 | 1 or 2 or 3 or 4 or 5 or 6 or 7 or 8 or 9 or 10 or 11 or 12 or 76 or 77 or 78 or 79 or 80 or 81 or 83 or 84 or 85 or 86 or 87 or 88 or 89 or 90 or 91 or 92 | escalation of care |
| 94 | 13 or 14 or 15 or 16 or 17 or 18 or 19 | patient centred approach |
| 95 | 20 or 21 or 22 or 23 or 24 or 25 or 26 or 27 or 28 or 29 or 30 or 31 or 32 | CMO 1 |
| 96 | 33 or 34 or 35 or 36 or 37 or 38 or 39 or 40 or 41 | CMO 2 |
| 97 | 42 or 43 | CMO 3 |
| 98 | 44 or 45 or 46 or 47 or 48 or 49 or 50 or 51 or 52 | CMO 4 |
| 99 | 53 or 54 or 55 or 56 or 57 or 58 or 59 or 60 or 61 | CMO 5 |
| 100 | 62 or 63 or 64 or 65 or 66 or 67 or 68 or 69 or 70 | CMO 6 |
| 101 | 70 or 71 or 72 or 73 or 74 or 75 | CMO 7 |
| 102 | 95 or 96 or 97 or 98 or 99 or 100 or 101 |  |
| 103 | 93 and 94 and 102 |  |
