# Supplementary material for: Examining how goals of care communication are conducted between doctors and patients with severe acute illness in hospital settings: A realist systematic review
Source: PLoS One. 2024 Mar 18;19(3):e0299933. doi: 10.1371/journal.pone.0299933 (PMC10947705; doi:10.1371/journal.pone.0299933)
Supplement: S2 Appendix — (DOCX) [file pone.0299933.s004.docx]

**Appendix 2: Quality appraisal**

**Qualitative studies**

| **Author, year** | **1** | **2** | **3** | **4** | **5** | **6** | **7** | **8** | **9** | **10** | **Score** | **% score given** | **Quality grade rating** |
| --- | --- | --- | --- | --- | --- | --- | --- | --- | --- | --- | --- | --- | --- |
| Ashana et al 2022 |  |  |  |  |  |  |  |  |  |  | 7 | 70 | Moderate |
| Bedulli et al 2023 |  |  |  |  |  |  |  |  |  |  | 6 | 60 | Moderate |
| Deep et al 2008 |  |  |  |  |  |  |  |  |  |  | 7 | 70 | Moderate |
| Deep et al 2008 |  |  |  |  |  |  |  |  |  |  | 6 | 60 | Moderate |
| Dzeng et al 2015 |  |  |  |  |  |  |  |  |  |  | 8 | 80 | High |
| Haliko et al 2019 |  |  |  |  |  |  |  |  |  |  | 7 | 70 | Moderate |
| Harris et al 2021 |  |  |  |  |  |  |  |  |  |  | 8 | 80 | High |
| Hayes, 2010 |  |  |  |  |  |  |  |  |  |  | 8 | 80 | High |
| Hutchison et al 2016 |  |  |  |  |  |  |  |  |  |  | 9 | 90 | High |
| Kryworuchko et al 2015 |  |  |  |  |  |  |  |  |  |  | 6 | 60 | Moderate |
| Lagrotteria et al 2021 |  |  |  |  |  |  |  |  |  |  | 8 | 80 | High |
| Levinson et al 2019 |  |  |  |  |  |  |  |  |  |  | 7 | 70 | Moderate |
| Lindberg et al 2015 |  |  |  |  |  |  |  |  |  |  | 8 | 80 | High |
| Lu et al 2015 |  |  |  |  |  |  |  |  |  |  | 7 | 70 | Moderate |
| Pham et al 2008 |  |  |  |  |  |  |  |  |  |  | 10 | 100 | High |
| Schonfeld et al 2012 |  |  |  |  |  |  |  |  |  |  | 8 | 80 | High |
| Shah et al, 2016 |  |  |  |  |  |  |  |  |  |  | 6 | 60 | Moderate |
| Sterie 2021 |  |  |  |  |  |  |  |  |  |  | 9 | 90 | High |
| Strachen et al 2018 |  |  |  |  |  |  |  |  |  |  | 9 | 90 | High |
| Sullivan et al, 1996 |  |  |  |  |  |  |  |  |  |  | 7 | 70 | Moderate |
| Taylor et al, 2018 |  |  |  |  |  |  |  |  |  |  | 8 | 80 | High |
| Uy et al 2013 |  |  |  |  |  |  |  |  |  |  | 6 | 60 | Moderate |
| Vanderhaeghen et al 2019 |  |  |  |  |  |  |  |  |  |  | 7 | 70 | Moderate |
| Wubben et al 2021 |  |  |  |  |  |  |  |  |  |  | 9 | 90 | High |
| Griffiths et al 2021 |  |  |  |  |  |  |  |  |  |  | 7 | 70 | Moderate |
| Rasmussen et al 2018 |  |  |  |  |  |  |  |  |  |  | 5 | 50 | Low |
| Eli et al 2021 |  |  |  |  |  |  |  |  |  |  | 8 | 80 | High |

**Joanna-Briggs Institute appraisal checklist for qualitative studies (https://jbi.global/critical-appraisal-tools)**

Is there congruity between the stated philosophical perspective and the research methodology

1. Is there congruity between the research methodology and the research question or objectives?
2. Is there congruity between the research methodology and the methods used to collect data?
3. Is there congruity between the research methodology and the representation and analysis of data?
4. Is there congruity between the research methodology and the interpretation of results

**Cohort studies**

| **Author, Year** | **1** | **2** | **3** | **4** | **5** | **6** | **7** | **8** | **9** | **10** | **11** | **Score** | **% score given** | **Quality grade rating** |
| --- | --- | --- | --- | --- | --- | --- | --- | --- | --- | --- | --- | --- | --- | --- |
| Ros et al, 2021 |  |  |  |  |  |  |  |  |  |  |  | 10 | 91 | High |

**Joanna-Briggs Institute appraisal checklist for cohort studies (https://jbi.global/critical-appraisal-tools)**

1. Were the two groups similar and recruited from the same population?
2. Were the exposures measured similarly to assign people to both exposed and unexposed groups?
3. Was the exposure measured in a valid and reliable way?
4. Were confounding factors identified?
5. Were strategies to deal with confounding factors stated?
6. Were the groups/participants free of the outcome at the start of the study (or at the moment of exposure)?
7. Were the outcomes measured in a valid and reliable way?
8. Was the follow up time reported and sufficient to be long enough for outcomes to occur?
9. Was follow up complete, and if not, were the reasons to loss to follow up described and explored?
10. Were strategies to address incomplete follow up utilized?
11. Was appropriate statistical analysis used?

**Randomised control trials**

| **Author, Year** | **1** | **2** | **3** | **4** | **5** | **6** | **7** | **8** | **9** | **10** | **11** | **12** | **13** | **Score** | **% score given** | **Quality grade rating** |
| --- | --- | --- | --- | --- | --- | --- | --- | --- | --- | --- | --- | --- | --- | --- | --- | --- |
| Lee et al 2022 |  |  |  |  |  |  |  |  |  |  |  |  |  | 10 | 77 | Moderate |
| Pollak et al 2019 |  |  |  |  |  |  |  |  |  |  |  |  |  | 7 | 53 | Low |

**Joanna-Briggs Institute appraisal checklist for randomised control trials (https://jbi.global/critical-appraisal-tools)**

1. Was true randomization used for assignment of participants to treatment groups?
2. Was allocation to treatment groups concealed?
3. Were treatment groups similar at the baseline?
4. Were participants blind to treatment assignment?
5. Were those delivering treatment blind to treatment assignment?
6. Were outcomes assessors blind to treatment assignment?
7. Were treatment groups treated identically other than the intervention of interest?
8. Was follow up complete and if not, were differences between groups in terms of their follow up adequately described and analyzed?
9. Were participants analyzed in the groups to which they were randomized?
10. Were outcomes measured in the same way for treatment groups?
11. Were outcomes measured in a reliable way?
12. Was appropriate statistical analysis used?
13. Was the trial design appropriate, and any deviations from the standard RCT design (individual randomization, parallel groups) accounted for in the conduct and analysis of the trial?

**Observational non-experimental studies**

| **Author, year** | **1** | **2** | **3** | **4** | **5** | **6** | **7** | **8** | **Score** | **% score given** | **Quality grade rating** |
| --- | --- | --- | --- | --- | --- | --- | --- | --- | --- | --- | --- |
| Andersen et al 2011 |  |  |  |  |  |  |  |  | 5 | 63 | Moderate |
| Carrard et al 2018 |  |  |  |  |  |  |  |  | 7 | 75 | Moderate |
| Deptola et al 2019 |  |  |  |  |  |  |  |  | 2 | 24 | Low |
| Syed et al 2017 |  |  |  |  |  |  |  |  | 6 | 75 | Moderate |
| Weigl et al 2009 |  |  |  |  |  |  |  |  | 4 | 50 | Low |
| You et al 2019 |  |  |  |  |  |  |  |  | 6 | 75 | Moderate |

**Joanna-Briggs Institute appraisal checklist for analytical cross-sectional studies (https://jbi.global/critical-appraisal-tools)**

1. Were the criteria for inclusion in the sample clearly defined?
2. Were the study subjects and the setting described in detail?
3. Was the exposure measured in a valid and reliable way?
4. Were objective, standard criteria used for the measurement of the condition?
5. Were confounding factors identified?
6. Were strategies to deal with confounding factors stated?
7. Were the outcomes measured in a valid and reliable way?
8. Was appropriate statitiscal analysis used?

**Expert opinion articles**

| **Author, year** | **Score** | **1** | **2** | **3** | **4** | **5** | **6** | **Score** | **% score given** | **Quality grade rating** |
| --- | --- | --- | --- | --- | --- | --- | --- | --- | --- | --- |
| Charles et al 2006 | 6 (100%) |  |  |  |  |  |  | 6 | 100 | Low |
| Loewenstein 2005 | 6 (100%) |  |  |  |  |  |  | 6 | 100 | Low |
| Tulsky et al 2017 | 6 (100%) |  |  |  |  |  |  | 6 | 100 | Low |
| Vitale et al | 5 (83%) |  |  |  |  |  |  | 5 | 83 | Low |
| Casteneda-Guarderas et al 2016 | 6 (100%) |  |  |  |  |  |  | 6 | 100 | Low |
| Kon et al 2016 | 6 (100%) |  |  |  |  |  |  | 6 | 100 | Low |
| Thomas et al 2021 | 6 (100%) |  |  |  |  |  |  | 6 | 100 | Low |
| Mentzelopoulos et al 2021 | 6 (100%) |  |  |  |  |  |  | 6 | 100 | Low |

**Joanna-Briggs Institute appraisal checklist for expert opinion articles (https://jbi.global/critical-appraisal-tools)**

1. Is the source of the opinion clearly identified?
2. Does the source of opinion have standing in the field of expertise?
3. Are the interests of the relevant population the central focus of the opinion?
4. Is the stated position the result of an analytical process, and is there logic in the opinion expressed?
5. Is there reference to the extant literature?
6. Is any incongruence with the literature/sources logically defended?

**Ethical argument papers**

| **Author, year** | **Score** | **1** | **2** | **3** | **4** | **5** | **Score** | **% Score given** | **Quality grade rating** |
| --- | --- | --- | --- | --- | --- | --- | --- | --- | --- |
| Dubov 2017 | 5 (80%) |  |  |  |  |  | 4 | 80 | Low |

**Jansen and Ellerton’s Ethics critical appraisal worksheet (Jansen M, Ellerton P. How to read an ethics paper. J Med Ethics. 2018 Dec;44(12):810-813. doi: 10.1136/medethics-2018-104997)**

1. What is the point at issue?
2. Has the author defined all of the terms they use?
3. Dissect the argument: (a) What are the premises of the author’s argument? (b) What are the author’s conclusions?
4. Does the author address all relevant counterarguments?
5. Is the argument or exploration of the issue relevant to your practice?

**Systematic reviews**

| **Author, year** | **1** | **2** | **3** | **4** | **5** | **6** | **7** | **8** | **9** | **10** | **11** | **Score** | **% score given** | **Quality grade rating** |
| --- | --- | --- | --- | --- | --- | --- | --- | --- | --- | --- | --- | --- | --- | --- |
| Brooks et al, 2018 |  |  |  |  |  |  |  |  |  |  |  | 8 | 73 | Moderate |
| Vanderhaeghen et al 2018 |  |  |  |  |  |  |  |  |  |  |  | 10 | 91 | High |
| Visser et al 2014 |  |  |  |  |  |  |  |  |  |  |  | 10 | 91 | High |

**Joanna-Briggs Institute appraisal checklist for systematic reviews (https://jbi.global/critical-appraisal-tools)**

1. Is the review question clearly and explicitly stated?
2. Were the inclusion criteria appropriate for the review question
3. Was the search strategy appropriate?
4. Were the sources and resources used to search for studies adequate?
5. Were the criteria for appraising studies appropriate?
6. Was critical appraisal conducted by two or more reviewers independently?
7. Were there methods to minimize errors in data extraction?
8. Were the methods used to combine studies appropriate?
9. Was the likelihood of publication bias assessed?
10. Were recommendations for policy and/or practice supported by the reported data?
11. Were the specific directives for new research appropriate?

**Mixed methods studies**

|  | Each section scored taking an average view on each of the 5 subcomponents | | | | | | | |
| --- | --- | --- | --- | --- | --- | --- | --- | --- |
| **Author, year** | **1** | **2** | **3** | **4** | **5** | **Score** | **% score given** | **Quality grade rating** |
| Hart et al, 2021 |  |  |  |  |  | 5 | 100 | High |
| Periyakoil et al 2015 |  |  |  |  |  | 3 | 60 | Moderate |
| Sharma et al 2014 |  |  |  |  |  | 1 | 20 | Low |
| Bristowe et al 2015 |  |  |  |  |  | 3 | 60 | Moderatre |

**Mixed methods appraisal tool (http://mixedmethodsappraisaltoolpublic.pbworks.com/w/file/fetch/127916259/MMAT_2018_criteria-manual_2018-08-01_ENG.pdf)**

S1. Are there clear research questions?

S2. Do the collected data allow to address the research questions?

**Qualitative**

1.1. Is the qualitative approach appropriate to answer the research question?

1.2. Are the qualitative data collection methods adequate to address the research question?

1.3. Are the findings adequately derived from the data?

1.4. Is the interpretation of results sufficiently substantiated by data?

1.5. Is there coherence between qualitative data sources, collection, analysis and interpretation?

**RCTs**

2.1. Is randomization appropriately performed?

2.2. Are the groups comparable at baseline?

2.3. Are there complete outcome data?

2.4. Are outcome assessors blinded to the intervention provided?

2.5 Did the participants adhere to the assigned intervention?

**Non randomiused studies**

3.1. Are the participants representative of the target population?

3.2. Are measurements appropriate regarding both the outcome and intervention (or exposure)?

3.3. Are there complete outcome data?

3.4. Are the confounders accounted for in the design and analysis?

3.5. During the study period, is the intervention administered (or exposure occurred) as intended?

**Quantitative descriptive studies**

4.1. Is the sampling strategy relevant to address the research question?

4.2. Is the sample representative of the target population?

4.3. Are the measurements appropriate?

4.4. Is the risk of nonresponse bias low?

4.5. Is the statistical analysis appropriate to answer the research question?

**Mixed methods studies**

- 1. Is there an adequate rationale for using a mixed methods design to address the research question?
  2. Are the different components of the study effectively integrated to answer the research question?
  3. Are the outputs of the integration of qualitative and quantitative components adequately interpreted?
  4. Are divergences and inconsistencies between quantitative and qualitative results adequately addressed

5.5. Do the different components of the study adhere to the quality criteria of each tradition of the methods involved?
