# Supplementary material for: Examining how goals of care communication are conducted between doctors and patients with severe acute illness in hospital settings: A realist systematic review
Source: PLoS One. 2024 Mar 18;19(3):e0299933. doi: 10.1371/journal.pone.0299933 (PMC10947705; doi:10.1371/journal.pone.0299933)
Supplement: S3 Appendix — (DOCX) [file pone.0299933.s005.docx]

**SUPPLEMENTARY FILE: STUDY/LITERATURE CHARACTERISTICS**

| **Author** | **Main study findings** | **Contribution to CMO** | **Relevant Extract** |
| --- | --- | --- | --- |
| Anderson et al 2011 | The median length of code status discussions was 1 minute (range 0.2-8.2)  Prognosis was discussed with code status in only 1 (out of 19) of encounters.  Discussions focused on the life-sustaining interventions as opposed to larger life goals  Descriptions of CPR as an intervention used medical jargon  The indication for CPR was framed in general, as opposed to patient-specific scenarios. | **INFORMATION:** In this study, code status discussion was very short (median length 1 min – range 0.2-8.2 mins); Prognosis was discussed on 1/19 encounters. Information focussed largely on medical interventions offered and not broader life goals.  If QoL was brought up by patients, physicians rarely asked patients to elaborate further (therefore not a personalised approach)  6/19 encounters mentioned All components of: risk, benefits and possible outcomes of CPR. “Death or Die” only mentioned (2/19); When describing CPR as an intervention, jargon such as “resuscitation,” “CPR,” and “compressions” was used but not defined.  Patients mentioned risks, benefits, and outcomes of CPR in terms of their status after a code, using euphemisms such as “vegetable” or “invalid.” Patients’ meanings of euphemisms were not explored  **INFLUENCE:** The indication for CPR was framed in terms such as “emergency” along with phrases such as “if your heart were to stop”  **CONFIDENCE/SKILLS:** In no conversations did physician provide recommendation | “PATIENT: My basic position has been that I do not want my life extended...[there is a] serious question as to whether I would be able to return to a relatively normal or natural life. I don’t want any artificial means taken to extend my life, if it’s not going to extend the quality of my life. PHYSICIAN: I see. I ask because some people absolutely don’t want anything done. And other people say, “Do everything.” And there’s a lot of gray in between. So I want to make sure we respect your wishes. But I think I have a sense of where you’re coming from |
| Ashana et al 2022 | Clinicians most frequently expressed difficulty discussing ACP with certain racial and ethnic groups (African American, Hispanic, Asian, and Native American) (31.1%), non-native English speakers (24.3%), and those with certain religious beliefs (Catholic, Orthodox Jewish, and Muslim) (13.5%). Clinicians were more likely to attribute barriers to ACP completion to patients (62.2%), than to clinicians (35.1%) or health systems (37.8%). Three themes characterized clinicians’ difficulty approaching ACP (preconceived views of patients’ preferences, narrow definitions of successful ACP, and lack of institutional resources), while the final theme illustrated facilitators to ACP (acknowledging bias and rejecting stereotypes, mission-driven focus on ACP, and acceptance of all preferences). | **INFLUENCE:** Patients making decisions relating to what their family want rather than according to their own values.  **TRUST/RELATIONSHIP:** Pre-conceived patient views or preferences - potentially hampered by bringing up ACP discussions as may not be true understanding of a patients cultural values and starting ACP discussion can have a negative impact.  Cultural differences and lack of cultural understanding and sensitivity by the doctor can result in conflict and discordant views and can erode trust  **SKILLS:** Acknowledging bias and rejecting stereotypes is a potential facilitator to ACP in marginalised groups.  Acceptance of all preferences even those who do not wish to engage or those who wish family member to speak on behalf of the patient:  **ORGANISATIONAL:** Many clinicians defined successful ACP by completion of forms, including specifying a surrogate decision maker and documenting medical directives. Conversely, not focussing on forms and more on the ACP process may be a facilitator to ACP conversations.  Finality associated with agreeing to ceiling of care decision/having something documented  Many clinicians cited insufficient institutional resources as a barrier to engaging structurally marginalized populations in ACP. They reported lack of in-person interpreters with training in discussing ACP, and limited availability of ACP documents in multiple languages or designed for users with limited health literacy. | “I have [Spanish-speaking] patients who... don’t want to burden their family. The decision to be DNR (do-not-resuscitate)... is counter to what their family’s expect- ation is for them. So they will choose to be full CPR. That is bewildering to me because it doesn’t seem consistent with their values.”  One said (female [F], physician), “[In] non-White cultures... there is this idea that if you talk about death, it’s going to make it happen.” Another participant (F) remarked, “In... Hispanic culture... you can’t tell grandma that she has cancer because she’ll give up and die.” Another (F, physician) remarked, “It is uncomfortable... to be accused, ‘You think you can undo God’s will’. If you get that often enough, you subconsciously are going to avoid that conversation again.” A female physician said, “We see people from many countries. We don’t know their practices. We might offend someone by asking.” ; One physician (F) shared, “I love my [African American] patients, but the minute that I bring up [dying], I feel this... hit the brakes, and I don’t know if it’s my own insecurities, but... it brings up... not trusting me.”  “[The patient’s wife] was very frustrated because... the [doctors] keep bringing the same things up, and she is very clear of her goals. And they are different from what... the [doctors] want her husband’s goals to be. It’s about cultural differences... I know spirituality was a large driver of what her preferences were.”  One said (F, physician), “It’s a bigger pitfall to make an assumption about... you’re Orthodox Jewish, so you have this belief. You have to leave the door open for everybody.” Another (M, chaplain) explained, “A Muslim from Indonesia... might think about medical decisions differently than Muslims from Kuwait. Realizing there is... these broad strokes of religious traditions... but it ultimately comes down to this family and this person because there is such a diversity of how they might apply it.”  A physician (F) noted, “Do you speak for you? Alright, I’m with you. On the other hand, if son is the one who speaks for you, I have to respect that as well.” A participant (M) said, “Some people... show love and care through... medical interventions. And culturally... their values are going to be different than mine and realizing it can make sense in those cases.” Another (F) described that successful ACP may result in respecting patients’ wishes to avoid ACP altogether, “Advance care planning tends to preference a world view that anticipates a future. That is very... autonomy and con- trol focused and doesn’t resonate with all people.”  A physician (M) said, “Part of my success with ACP conversations is... that I can do them really fast. I don’t think it has to be [a conversation]. I can do an MDPOA (medical durable power of attorney) using [my state’s] form in 30 seconds.” A participant (F) explained, “Our objective is to check the box, have a document that we can scan into the medical record.”  “[Hispanic patients] think when they sign that piece of paper... they no longer have a choice and it’s kind of like a sign of giving up.”  “We have... an iPad virtual translator. Sometimes the volume isn’t loud enough. It’s hard to read the body language. We used to have in-person translators, and that was... better.” Another physician (M) remarked, “There’s a state website on ACP. But that’s not available in Spanish, and half of our patients are Spanish speaking.” A partici- pant (F) shared, “In Texas, we have a lot of Spanish-speaking folks, but you [cannot] print the DNR in Spanish. Any legal document has to be in English.” |
| Bedulli et al 2023 | On the one side, CPR/DNAR decisions were dominated by the belief that patient involvement is often pointless, even though participants favoured a shared decision-making approach. On the other, despite aiming at a non-manipulative conversation, participants were aware that most CPR/ DNAR conversations are characterized by a nudging communicative approach where the physician gently pushes patients towards his/her recommendation | **INFORMATION PROVISION:** Doctors acknowledging importance of checking patient understanding, expectation, wishes and values  Not personalised  **INFLUENCE:** Acknowledgement that remaining neutral, factual and non-leading information is important.  Leading conversations  **TRUST/RELATIONSHIP:** Important to establish rapport BEFORE the GoC conversation takes place.  Desire to preserve patient’s hope and patients alliance by not engaging.  Saying that something can always be done  Establishing rapport and trust really important.  Feeling of abandonment is a risk to goals of care planning  Feeling that any decision is “set in stone” may negatively impact relationship.  Tailor the communication style to the patient  Being personable/showing humility  Taboo around death  Cultural aspect relating to the value of life  **CONFIDENCE/SKILLS:** During the conversation, they reported the need to provide neutral and factual information that does not lead but rather guides the patient; inform patients and their families, in case of an unilateral DNAR order, that this does not preclude optimal care or treatment of the underlying condition; stress that the decision is not irreversible and can be adapted across conditions, not only with patients and their families, but also among the care team; keep the conversation open, avoiding the tendency to fit every answer to either a “yes” or “no”; consider the larger social context of the patients and their emotional burden; use words that communicate presence; tailor the communication style to the patient; show humility; and embrace the conversation with courage and truthfulness. At the end of the conversation, participants reported the need to sum up its content, particularly the process through which a CPR/DNAR decision was reached.  To solve this tension, participants suggested to offer timely communication training to physicians, have a dedicated communication professional in the team, ensure physicians are exposed to as many CPR/DNAR conversations as possible and offer debriefing sessions  The need for timely communication skills training  Exposure to conversations and debriefing  Reassure patients that conversations are part of the routine  Give patient time to process information  **VALUE:** Many HCPs agree advanced discussions are helpful  Talking about DNACPR in the context of broader planning  Involvement of the patient in the decision-making is futile until proven helpful.  Presumed patient lack of capacity  Presumed poor prognosis is a barrier to discussions  Young/good health also a barrier  Desire to avoid scaring the patient  **ORGANISATIONAL:** To solve this tension, participants suggested to offer timely communication training to physicians, have a dedicated communication professional in the team, ensure physicians are exposed to as many CPR/DNAR conversations as possible and offer debriefing sessions, increase involvement of GPs and specialists to promote these conversations outside of the hospital setting, reassure patients and their families that the conversation is part of routine and that they are not alone, assess patient’s interpretations of the information received, and give the patient time to elaborate the information  Importance of holding the conversations in the right place  No appropriate location to have the conversation  No time to have effective conversations  Support having these conversations  Hospital ACP policy may not be helpful: | “We need to focus on the patient’s objectives, not only on the therapies that we doctors want [. . .] What their values are, what their importance is, even if the patient, at that moment, is not in a phase of illness. So that they can say what they want, what their life is, and what they expect from an illness, and on this we decide a priori, before the illness, we decide what will be done if the illness arrives, without waiting. Every day [we should] have on our board, on our patient’s board, their goal —which is not ours, and which is detached from the medical act. (FG4)”; “I say, ideally, we should start, in a situation where we have to understand how far to go with the treatments. The first thing is to understand what the patient has understood about their situation and their pathology, so we can explain them a little, and get on their level, and only then get to the problem we have to solve together. (FG2)”  “We are not so attentive to the needs of the patient because we, too often, focus on treating the disease and not on treating the person. (FG4)”  “One must remain neutral. Explain things correctly. (FG1); “We should never get involved in the decision. We can explain to them, we can reassure them, but the important thing is never to influence them. (FG1)”  “It is us who ultimately make the family member choose what we would have done, so we have to acquire this awareness that we are the ones who decide. Depending on how I present the possible outcome of resuscitation or admission to the intensive care unit, or how I present the development, I end up putting words in the mouth of the family members who tell me not to do it…We should be aware of this role and power we have, particularly when we lead a discussion about advance directives in an urgent context. (FG2)”  “I would rather wait a few days so that there is more of a relationship of trust there, and then the patient may ask “doctor, what would you do?”. And that makes things much easier. (FG1)”; For me it is very important that a therapeutic relationship has already been established with the patient”. (FG3)  “Perhaps it is because of a fear on the part of the physician to extinguish the patient’s hope in treatment and in the possibility of being able to do something about their illness…They may find it hard to extinguish [the patient’s] hope in this way, hard to tell the patient the way things really are….In my opinion this holds back a little bit the physicians, this fear of losing the therapeutic alliance with the patient. (FG4)”  “[We should] re-establish the therapeutic alliance by re-establishing the goal according to the current condition, informing them that we are there and that something can always be done with the goal perhaps no longer to heal and return to life as before, but with the goal of living an acceptable quality of life”  “I would rather wait a few days so that there is more of a relationship of trust there, and then the patient may ask “doctor, what would you do?”. And that makes things much easier. (FG1)”; “For me it is very important that a therapeutic relationship has already been established with the patient. (FG3)”  “They may think we just let them die like that, but that is simply not what comes next. (FG1)”; It is frustrating that they may think we want to abandon them. (FG2)  “what also stops the patient and the families a bit is the feeling of irreversibility of the choice. The choice is made and from that moment there can be no more discussion”  “You also have to adapt to the moment, it’s not that there is a right formula for everyone, just how you get feedback from relatives and family members. Because you can’t use the same words with everyone, sometimes you have to be as sweet and empathetic as possible while sometimes you need to use the bluntest words for the message to get through. (FG1)”  “I think it is also important to be humble [. . .] Finally we talk about death and life and the patient is not just a patient and has a whole life before they were ill. (FG3)”  “There is a cultural aspect, I have never lived in a culture where it was easy to talk 110 about death, I don’t know if there are any. Sometimes we are surprised to see elderly patients and they have never spoken about it. (FG2)”  It was precisely in this context that I had found a cultural aspect that was linked to a component of religion, even if less and less practised, this Catholic component of the Southern Alps I had felt very strongly. As also a certain concept of the sacredness of life and therefore one could help lengthen it. I had really felt it from a socio-cultural and religious point of view. (FG4)”; Society nowadays no longer accepts that someone dies, let’s say that no one would have asked fifty years ago to resuscitate an 80-year-old whereas now they would want to live another thirty, without considering the quality of life. . . And one who is not in the field of medicine does not imagine the post resuscitation and therefore I find that time spent explaining, in simple words, the complications often helps to find a solution. (FG4)  “It is not something you improvise. It’s something you have to learn how to do, there are communication techniques, there are communication strategies, there are also principles of law that you have to know and I think you need training. It should be part of the practice, before you start working, you have to, if we want to make some cultural change, you have to start fromwho has the power to direct families and patients. Sometimes it’s just a technical question on how to communicate and knowing what to say, what not to say, the phrases to use, the way to present oneself, or let someone speak. It is something you learn. (FG2)”  “The first few times I participated and kept my mouth shut, I listened and I also needed to see several people do it to see what techniques one and the other used. “Okay, this sentence I like a lot, okay, this example I will use it”. Then at the end one builds up one’s own knowledge…After the discussion, the person who had led the discussion, the conversation, would stop with me and we do a kind of debriefing where we analyse how the patient reacted, what we could say better. It is really a structured thing. It has to be kind of spread out in a structured way. (FG2)”  “Then you start by saying, “we would like to talk to you about something, it’s an issue we deal with all the patients we hospitalise”. You really try to anticipate the fact that we are not in an emergency situation but that these are issues we deal with routinely in our activity. (FG1)”  “[We should] ask questions, yes, but also try to wait. I have noticed from palliativists that they ask a question and then keep quiet, which I have never done, and after five or ten seconds the patient starts to open up. Here, don’t be in a hurry to get an answer, ask the question and leave time to answer”  “Discussing it now and not waiting until the last moment to do things like advance 2 directives regarding the patient’s conception of life. These things should be done as early as possible, or at least introduced”  “It makes more sense to me to say “Let’s go beyond the current hospitalisation and 6 problem, and let’s talk about what is more long-term planning. Let us talk more broadly about the planning of a person’s health”. (FG2)  “I also think about the imminence. . . I mean, whether it is useful to talk about it as soon as possible with a patient with respiratory distress and who might shortly have an arrest. If, on the other hand, it is a patient who has a team following them, and is expected to be taken care of, then one can wait with common sense. (FG3)”  “There are decisions that patients are not able to make because only a doctor can understand what the consequences for the patient might be. And so we risk to overtreat and do futile treatments”. (FG1); If they don’t get there, it is useless. And at that moment we need to talk to the family doctor and share the choice with them, and if they don’t have the tools, the education, at most the choice has to be imposed and then shared”; “If one has very clear ideas, it is very easy, and no time is wasted. If, on the other hand, you have to start from scratch and there are no tools, there is no point in wasting time and one has to impose oneself and give the indication, perhaps sharing it with the family doctor if the choice is a bit borderline. (FG2)”; “People wouldn’t understand it, they don’t have the tools to understand it in a context that seems totally unconnected to them. It’s different in the hospital in Zurich where they are asked "do you want to be resuscitated or not?", there’s a different culture, so you expect to be asked [that question]. (FG2)”; “It’s so bad to leave the decision in the hands of a 90-year-old where they don’t even know what I’m talking about, they can’t even imagine. Where they tell me that they want us to do everything for them. In that case, I learned to take the relatives aside, and to explain to them what resuscitation means to a patient of that age, and how they would come out of it [CPR]” “Perhaps, at certain times, the patient is unable to decide for themselves, and 21 therefore there is no point in discussing this with the patient if it is not possible. (FG1)”  “It also happens in everyday hospital life not to discuss the advance directives with the patient. It has happened to all of us to say “OK, this is a patient who, if they have a complication, is not resuscitated, by medical choice, because he has a number of impairments and pathologies that would not give them a chance of a good recovery”. And so normally you inform them that you established this attitude of care, but we do not always discuss it with the patient. (FG1)”  With a healthy patient it may be difficult to deal with such talk (FG4)”  “It’s really pointless because you know the situation is so bad. . . Why scare them further?”; “If they have never thought about it and they come to the emergency room, we don’t have time to have a good discussion, we just create anxiety in the patient”  “Of course it should not be done in a room with four other patients. You have to find a secluded place or a quiet room where no one will disturb you. You leave the phone with your colleague and ask the nurse not to disturb you unless it is strictly necessary. (FG1)”  “It is not always guaranteed. There are departments where there is no physical 76 possibility to have such a discussion, not to be disturbed by the telephone.”  “A key factor is time, to explain to the patient and to ask. Time is crucial. The problem with doing it on the ward is that there is no such time because it can take hours and is often impossible. (FG3); ” It is easier to treat the disease and not the person and this takes much less time and 84 energy, and it is easier because there is often a defined pathway. (FG 4)”  “There should always be a figure who accompanies you, like a clinic leader or even a dedicated figure who comes, helps you along the way. If not, you really struggle. (FG3)”  What bothers me personally is that I always have to put a label on it, I don’t know if it happens to you too? Almost immediately, from the moment the patient comes in it is true that the patient can rush in but I am not like that sometimes, it becomes an automatic thing but the directives of a patient are not so trivial to have to decide in a short time, as we often do, because it is part of the things to do when a new patient comes in. (FG1)” |
| Bristowe et al 2015 | The AMBER care bundle was associated with increased frequency of discussions about prognosis between clinicians and patients (χ(2) = 4.09, p = 0.04), higher awareness of their prognosis by patients (χ(2) = 4.29, p = 0.04) and lower clarity in the information received about their condition (χ(2) = 6.26, p = 0.04). | **IDENTIFICATION:** Using a bundle (complex intervention) as a trigger tool for discussions has the potential to improve the frequency of such discussions but does not improve the quality of information discussed or communication (?without consideration given to the other components of this realist synthesis) |  |
| Brooks et al 2018 | Nurses identified that nurses and physicians were often uncomfortable and avoided communication about end-of-life care and prognosis.  Clinician and nurses roles: Physicians were seen to have a central or leading role, including coordinating communication with families including conveying prognosis and seeking consensus on resuscitation decisions; nurses perceived their role to be the ‘support person’, prompting physicians to communicate with the family, being a support person for families during con- versations, and fostering and enabling families to participate in decision-making. Nurses also described how their role involved cultural assessment to assess a family's religious needs. The level of nurse involvement in end-of-life communication was often dependent on the individual physician leading the communication. Nurses also perceived that physicians did not value nurses' opinions in communication, unless they were considered experienced and skilled in caring for ICU patients at the end-of-life, even though nurses generally wanted to have greater involvement.  Communication challenges in 7/9 studies: shift changes limiting ability to build rapport; not explaining poor prognosis in a culturally sensitive way often resulted in discordance in views potentially prolonging suffering and pain  Knowledge deficit: Clinicians frequently demonstrated knowledge deficit about culturally sensitive communication (including culture, race, ethnicity and religion; Knowledge deficit led to inadequate opportunities for communication with families and misunderstandings  Cultural influences on communication: clinicians' personal cultural values and religious beliefs and sociocultural characteristics responsible for avoiding conversations. This also includes sociocultural beliefs and practices related to their professions (e.g. medics v surgeons), and the ethnic, cultural, and religious background of patients and families. Potentially less ideal when. Families of culturally diverse backgrounds lacked awareness at times of how to communicate their cultural needs with clinicians, further increasing the risk of conflict  The cultural diversity of families may also contribute to unrealistic expectations related to end-of-life care. Families' unrealistic expectations in terms of prognosis or treatment due to cultural or religious reasons were at times overly optimistic regarding their relative's prognosis, which resulted in conflict between clinicians and families, and is- sues related to exchange of medical information | **INFLUENCE:** clinicians' personal cultural values and religious beliefs and sociocultural characteristics responsible for avoiding conversations. This also includes sociocultural beliefs and practices related to their professions (e.g. medics v surgeons; The cultural diversity of families may also contribute to unrealistic expectations related to end-of-life care. Families' unrealistic expectations in terms of prognosis or treatment due to cultural or religious reasons were at times overly optimistic regarding their relative's prognosis, which resulted in conflict between clinicians and families, and is- sues related to exchange of medical information  **TRUST/RELATIONSHIP:** Families of culturally diverse backgrounds lacked awareness at times of how to communicate their cultural needs with clinicians, further increasing the risk of conflict  **SKILLS:** Nurses identified that nurses and physicians were often uncomfortable and avoided communication about end-of-life care and prognosis. Clinicians frequently demonstrated knowledge deficit about culturally sensitive communication (including culture, race, ethnicity and religion; Knowledge deficit led to inadequate opportunities for communication with families and misunderstandings. Not explaining poor prognosis in a culturally sensitive way often resulted in discordance in views potentially prolonging suffering and pain; Education opportunities should focus on increasing clinicians' knowledge related to cultural awareness and building therapeutic relationships to support culturally diverse patients and families at the end-of-life  **ORGANISATIONAL:** Clinician and nurses roles: Physicians were seen to have a central or leading role, including coordinating communication with families including conveying prognosis and seeking consensus on resuscitation decisions; nurses perceived their role to be the ‘support person’, prompting physicians to communicate with the family, cultural assessment to assess cultural needs and being a support person for families during conversations, and fostering and enabling families to participate in decision-making; . Nurses also perceived that physicians did not value nurses' opinions in communication, unless they were considered experienced and skilled in caring for ICU patients at the end-of-life, even though nurses generally wanted to have greater involvement; shift changes limiting ability to build rapport. |  |
| Carrard 2016 | The more physicians displayed non-verbal dominance behaviours with patients who preferred dominance, the more favourable the consultation outcome | **TRUST/RELATIONSHIP/SKILLS:** In addition to clinical understanding of the patient, positive consultation outcomes are also associated with interpersonal accuracy (an individual’s ability to make correct inferences about others internal states, traits or personal attributes (e.g. the ability to recognise emotions, motivating factors or thoughts in others) and physician behavioural adaptability/flexibility - the tailoring of verbal and non-verbal elements of communication that is more suited to the communication style and preference of the patient. |  |
| Carrard et al 2018 | For female physicians, there was a significant positive link between interpersonal accuracy and both verbal and nonverbal behavioural adaptability (marginally significant for verbal). A different pattern was observed for male physicians with higher interpersonal accuracy skills being significantly related to less nonverbal behavioural adaptability. No significant link was observed between male physician interpersonal accuracy and their verbal behavioural adaptability.  female physicians who adapted their nonverbal (but not their verbal) behaviour had patients who reported more positive consultation outcomes. Moreover, the more female physicians were accurate interpersonally, the more they showed verbal and nonverbal behavioural adaptability. | **TRUST**: Behavioural adaptability is correlated with higher patient satisfaction and trust in female subset in this study (males were not explored as they generally did not display behavioural adaptability)  **SKILLS/CONFIDENCE:** Emotional intelligence: Males physicians are better than females and “reading” patients states/personality traits but generally do not adapt how they approach the patient based on this information.  Females, whilst not as good at “reading patients” adapt their non-verbal behaviour accordingly which is associated with more positive consultation outcomes (higher satisfaction and trust). The better they are at reading patients the more adaptability they show |  |
| Casteneda-Guarderas et al 2016 | Shared decision making (SDM) presents special challenges when used with vulnerable population groups. The differing circumstances, needs, and perspectives of vulnerable groups invoke issues of provider bias, disrespect, judgmental attitudes, and lack of cultural competence, as well as patient mistrust and the consequences of their social and economic disenfranchisement  Research agenda: 1) What are the best processes/formats for SDM among racial, ethnic, cultural, religious, linguistic, social, or otherwise vulnerable groups who experience disadvantage in the healthcare system? 2) What organizational or systemic changes are needed to support SDM in the ED whenever appropriate? 3) What competencies are needed to enable emergency providers to consider patients’ situation/context in an unbiased way? 4) How do we teach these competencies to students and residents? 5) How do we cultivate these competencies in practicing emergency physicians, nurses, and other clinical providers who lack them | **INFORMATION:** For those with low health literacy, important to provide the right amount of information without being condescending; Limited English proficiency: unavailability of appropriate translators in ED settings. Ideally, SDM materials and decision aids should be specifically developed for each minority/vulnerable patient group  **INFLUENCE:** Implicit bias (bias without being consciously aware) can affect information sharing, deliberation/recommendation, and decision making. Assumptions about class and education may also limit provider’s information sharing, with ethnic minority patients being less likely to report doctors discussing clinical experience and research; Culture and religion: People from different cultural and religious backgrounds may have differing norms, values, patterns of communication, beliefs about health and illness, and approaches to decision making. Physicians require cultural competency when conducting SDM in vulnerable populations; Limited English proficiency: unavailability of appropriate translators; LGTB: inherent biases toward patients may impede information exchange, which may result in ignored evidence, preferences, or general information  **TRUST/RELATIONSHIP:** Provider behaviour as the result of implicit bias or stereotyping can adversely affect patient trust in minority patients. Patients may also hold beliefs, attitudes or stereotypes towards providers, independent of provider behaviour that advserely affects provider-patient communication. May be most prominent with those with low health literacy; Individuals who are uneducated, unemployed, uninsured, undocumented, or undomiciled have increased ED healthcare utilsation. Judgmental attitudes from providers, even if not explicitly expressed, negatively affect physician–patient trust. Conversations may also be inhibited by a perceived power differential between physicians and their patients, beyond that experienced by more affluent patients; LGBT: Lack of knowledge and/or experience treating these individuals could potentially translate to provider attitudes of disapproval or discomfort that impede or prevent attempts at using SDM; Other threats to trust: disease stigma for: HIV/AIDS, psychiatric illness, substance abuse, anorexia and bulimia, sickle cell anaemia, and certain physical disabilities.  **SKILLS/CONFIDENCE:** Physicians need specific culture awareness and competency training and aware of applying SDM to those in minority groups by being non-judgmental and maintaining respect and developing trust and good rapport. This would empower patients to engage in SDM processes and reduce inequalities.  **ORGANISATIONAL**: organisations need to be culturally aware and sensitive |  |
| Charles et al 2006 | Cultural values/core belief systems can influence how patients make treatment decisions and their preferred treatment choice.  Cultural expectations will influence the nature of the encounter and how it proceeds, e.g. who is involved, their status, beliefs, role expectations (norms of interaction) and behaviour  Cultural influences can also help HCP see our own treatment decision-making behaviour, treatment preferences and criteria and reflect that these may not be shared by others outside their our own cultural group. Specific barriers to and facilitators of patient participation in treatment decision-making may also vary across different cultural groups.  Some potential sources of differences in perspective between physicians and patients that can influence their ideas and preferences about how to make treatment decisions include:   - Illness representations: ideas about the meaning and causes of illness; reactions to illness - Constructions of risk; patient values around benefits and risks of various treatments; concepts of risk and ways of assessing risk. - Treatment effectiveness: what counts as a good outcome; the role of biomedical science versus other influences/healers. - Treatment decision-making approaches: the meaning of a shared process of decision-making and how to implement it; the amount and type of information that patients want to know about their disease in order to make a decision; who should be involved in making the treatment decision and their preferences for involvement.   Family influence: In some cultures, multiple family members may have as much or more influence on the decision than the patient [27,33]. In other cultures there may be only one culturally prescribed decision-making approach which is considered acceptable rather than several options  Decision aids have not been designed with different cultural groups in mind and may not even be accepted by some cultures.  Values clarification exercises tend to be simplistic. A major problem with current exercises is that they neither encourage nor provide a mechanism for cultural values to be considered as legitimate factors in making a treatment decision | **INFORMATION:** Decision aids have not been designed with different cultural groups in mind and may not even be accepted by some cultures.  **INFLUENCE:** Cultural values/core belief systems can influence how patients make treatment decisions and their preferred treatment choice. Family influence: In some cultures, multiple family members may have as much or more influence on the decision than the patient. In other cultures there may be only one culturally prescribed decision-making approach which is considered acceptable rather than several options. Some potential sources of differences in perspective between physicians and patients that can influence their ideas and preferences about how to make treatment decisions include:   - Illness representations: ideas about the meaning and causes of illness; reactions to illness - Constructions of risk; patient values around benefits and risks of various treatments; concepts of risk and ways of assessing risk. - Treatment effectiveness: what counts as a good outcome; the role of biomedical science versus other influences/healers. - Treatment decision-making approaches: the meaning of a shared process of decision-making and how to implement it; the amount and type of information that patients want to know about their disease in order to make a decision; who should be involved in making the treatment decision and their preferences for involvement.   **TRUST/RELATIONSHIP:** Cultural expectations will influence the nature of the encounter and how it proceeds, e.g. who is involved, their status, beliefs, role expectations (norms of interaction) and behaviour. Cultural influences can also help HCP see our own treatment decision-making behaviour, treatment preferences and criteria and reflect that these may not be shared by others outside their our own cultural group. Specific barriers to and facilitators of patient participation in treatment decision-making may also vary across different cultural groups.  **CONFIDENCE/SKILLS:** Values clarification exercises tend to be simplistic. A major problem with current exercises is that they neither encourage nor provide a mechanism for cultural values to be considered as legitimate factors in making a treatment decision  **ORGANANISATIONAL:** Not explored here but organisations need to make cultural adaptations to ensure a fully equitable healthcare system across all cultures |  |
| Deep et al 2018 | Physician approach to the discussion   - Depersonalized disclaimers - Use of scripts   Patients and families lack adequate understanding   - Misconception of - resuscitation - Distinction between CPR, life support and vegetative state   Physicians question the decision   - Unreasonable expectations – - Preferences appear inconsistent   Physicians withhold guidance   - Not physicians’ responsibility - Respect agency and autonomy - Neutralize input from family members   Physicians implicitly influence decision making   - Normative framing - “Shock” talk   Balancing roles and responsibilities | **INFORMATION**  Information often delivered in a scripted, depersonalised and procedure-focused manner.  Patients/families had inadequate understanding of the decisions they were asked to make. This leads to inconsistencies and contradictions in what patients/families ask for  **INFLUENCE**: Three residents felt the input of a family member complicated the decision making process and inhibited them from sharing their medical recommendation for the patient.  Framing: Although many physicians denied explicitly influencing individuals in these decisions, the manner in which they framed the discussion of resuscitation was not value-free or without bias. This influence manifested in two ways in these discussions – “what we normally do” and “the alternative” and the “shock talk” where examples of graphic talk that would be perceived as highly invasive and off-putting e.g. “pounding” on the chest, putting “tubes down the throat”, “use electricity” to do “shock treatments” and “shooting the heart with medication | Including “medications that we can give to increase your heart rate, decrease your heart rate, or get it out of an irregular rhythm," “electrical paddles," and “pounding on their chest.”  “She wants all measures taken as possible, but if (she) has to stay alive on the machine, (she) don’t want to live on the machine. But if she can go on the machine like a ventilator and then come back off, she’s all for it”… and “They made it clear that (the patient’s) mom had been on a breathing machine for a long period of time and that (the patient) didn’t want to have that, but then she wanted everything done. So, it was...kind of contradictory.”  “I felt (the patient’s) daughter was taking an active part in the conversation, and she was encouraging her mother to say yes, is what I would say. So I felt like I almost had to be a mediator, you know, the neutral party because I’m sure if it were my mother I would probably want her to say yes too.” |
| Deep et al 2008 | In 6 dyads (21%), the participants reported i) differing interpretation of discussion. 2 pts had no recollection of discussion; 2 pts who wanted CPR were made DNACPR and 2 pts who requested DNACPR didn’t get the order  ii) Physicians misconstrues patient’s wishes  iii) Interference from family member  iv) fluctuating preferences based on information provided | **INFORMATION:** In 6 out of 28 dyads, there were discrepancies about the outcome of the discussion. 2 patients had no recollection of discussion despite passing cognitive screening test, in 4 patients the preferences of patient and physician differed. Two patients who desired resuscitation had orders limiting their care because the physician interpreted the discussion differently. Two patients who did not want resuscitation lacked DNR orders. Also, because of incomplete understanding. Patients exhibited fluctuating preferences  **INFLUENCE:** The involvement of a family member in the discussion led to a doctor–patient discrepancy when the patient and his spouse expressed different preferences. | Patient: ‘‘Just let me go. . . I just don’t want any life support.’’ Wife: ‘‘Naturally if your heart quits beating, they asked if you want revived. I would want him revived, but not put on life support.’’ Interviewer (to patient): ‘‘And you would not want to be revived?’’ Patient: ‘‘I don’t think so.’’ At various times throughout the interview, the patient’s spouse responded on behalf of the patient or interjected her preferences about his care |
| Deptola et al 2019 | Patients included in the improvement intervention were significantly less likely to transfer from the floor to an ICU (P= .005) and were significantly more likely to have a documented goals-of- care discussion during their inpatient stay (P<0.001)  There was a nonsignificant decrease in timing of GOC conversation documentation, from day 4.24 before intervention to day 3.64 during intervention (P= 0.321), for those patients admitted initially to the floor  There was a higher likelihood of dying outside the hospital as opposed to dying while admitted to a hospital for those who died within 1 year and received the intervention | **IDENTIFICATION:** Having an email alerting system in this study showed increased GoC discussions. Multiple potential study bias (including not tracking which emails were “picked up” and non-blinding means can’t be recommended to universally adopt but hypothesis generating and worth exploring in future studies. |  |
| Dubov 2015 | Debates whether critical care clinicians have a duty to persuade patients. This duty is supported by the fact that doctors often influence patients’ and families’ choices by framing presented options. Doctors have a duty of recognising and understanding these factors.  Persuasion differs from coercion, manipulation and deception.  Considering the fact that patients and families often make irrational decisions and the fact that doctors inadvertently influence their choices, the article suggested that persuasion can be a positive tool in medical communication. When patients or families clearly do not understand the risks or make decisions that contradict their long-term goals, persuasion can be used as a positive influence. | **INFLUENCE:** Persuasion in decision making may be appropriate as long as clinician recognises and is aware of this. Reasons why appropriate: clinician – vast clinical experience and superior knowledge base/extensive training vs patients with low health literacy who may not fully understand risks; limits the influence of highly charged emotions which can impact on making decisions which contradict their long term goals. | **INFLUENCE:** Persuasion in decision making may be appropriate as long as clinician recognises and is aware of this. Reasons why appropriate: clinician – vast clinical experience and superior knowledge base/extensive training vs patients with low health literacy who may not fully understand risks; limits the influence of highly charged emotions which can impact on making decisions which contradict their long term goals. |
| Dubrov 2017 | 2 models to describe risk perception and decision making in critical care:   - Intuitive – less “cognitive” based on rules and prior experiences - Analytical based on all the information available.   Intuition often takes significance due to the urgent nature that risk communication and decisions need to be made.  Emotions, intuitions and heuristics can all influence ethical thought and behaviour. Sometimes this leads to irrational thoughts from patients/families that do not necessarily align with values.  Intuition is subject to a host of cognitive biases relating to communication (e.g. loss/gain framing bias), availability, sunk-cost effect, order effect, bandwagon effect, default bias, omission bias, impact bias, representative bias  Moral heuristics also influence critical care decision making (see notes)  These take on more significance for situations that are either uncertain or ambiguous  Shaping patients or families decisions is an inescapable reality. Nudges or persuasion can be seen as a positive influence with strong ethical rationale because it steers decisions to those that align with a patients overall values whilst still allowing a degree of autonomy.  Doctors only rarely solicit patient values  Preferred model of autonomy: asking doctors to learn of the patient’s values but only to assist in matching those values with the medical facts of the situation. | **INFLUENCE:** Bias (both cognitive and non-cognitive) is unavoidable in SDM – particularly when intuitive decision making is practiced. Emotions, intuitions and heuristics can all influence ethical thought and behaviour. Sometimes this leads to irrational thoughts from patients/families that do not necessarily align with values. Intuition is subject to a host of cognitive biases relating to communication (e.g. loss/gain framing bias), availability, sunk-cost effect, order effect, bandwagon effect, default bias, omission bias, impact bias, representative bias. These take on more significance for situations that are either uncertain or ambiguous. Shaping patients or families decisions is an inescapable reality. Nudges or persuasion can be seen as a positive influence with strong ethical rationale because it steers decisions to those that align with a patients overall values whilst still allowing a degree of autonomy. Doctors only rarely solicit patient values. Preferred model of autonomy: asking doctors to learn of the patient’s values but only to assist in matching those values with the medical facts of the situation. |  |
| Dzeng et al 2015 | In hospitals with more autonomy focussed policies, autonomy was generally seen to be to over-arching ethical principle  Experienced physicians at all sites similarly did not exclusively allow their actions to be defined by policies and institutional culture and were willing to make recommendations against resuscitation if they believed it would be futile.  In contrast, less experienced are less inclined to make recommendations in autonomy based hospitals | **INFLUENCE:** Re-analysed Dzeng et al 2018 using Habermans theory of communicative action. Conversations are “distorted” to lead patients towards their desired goal or what they feel is in patients best interest (often subconsciously but also conscious efforts). More prevalent in autonomy-focussed hospitals. This may involve framing conversations using aggressive language to manipulate the patient/surrogates perception of actual choice.  **ORGANISATIONAL:** less experienced doctors (trainees) at hospitals where policies reflected autonomy focussed approaches often interpreted policies to mean that they were obliged to offer a choice of resuscitation in all cases regardless of patient’s likelihood of survival and they should not provide recommendations  This is in contrast to more experienced physicians were are not as influenced by policies and institutional culture. Extract from attending physician at autonomy focussed hospital | “For patients that I think should be DNR, I go into graphic detail pretty aggressively that we can do chest compressions which can break ribs and puncture lungs, which can be very painful, and we can put them indefinitely on a machine that could prolong their life without improving their quality of life. Then I usually say, ‘but of course it is your decision and it should be what you think they would want”  “I won’t get into representing how sick you are, but instead say, ‘would you like us to pound on your chest and break your ribs.’ They are infusing it with such aggressive language that there is a right answer. . .and it’s potentially not an accurate way to frame it. . .It is so laden with bias that you’re taking away the patient’s autonomy but still have the illusion of giving full autonomy to them. .”  “I think we face [futile CPR] a lot. I think if that’s what they want, that’s what they want. I think it’s a fair thing ... if they want it after understanding all of those things, then that’s what they want. I think to some extent, that it’s not our job to dictate what exactly what you should do ... it’s their decision. It’s their life, their body, they should choose whatever they want to be done with it.”  “If the patient gets worse, you’re going to hear me recommend DNR because I’m not too sure that doing chest compressions will actually save this person’s life.” |
| Eli et al 2021 | ReSPECT conversations usually were triggered when acute sudden clinical deterioration/immediate risk of dying or cardiac arrest or sometimes when a clear defined EoL diagnosis obtained; other trigger factors – organisational prompts or prompts from patient or family member. Promps from family member often when they were dissatisfied with not having any interaction with medical team and not knowing what’s going on  Most conversations were limited to CPR discussions with only a few going beyond this (e.g. ICU or other treatments e.g. Abx – particularly in palliative care conversations). Reason for avoidance related to not being immediatey relevant to the patient and concerns over information overload “The main reason for this is I didn’t want to overwhelm him, you know. ( . . . ) I wasn’t sure he was able to understand what ICU might have meant or all this sort of things. But he’s [an elderly patient] who is stable and going home today, so it’s very unlikely that he might need escalation... (Site 4, C03J)”  In most conversations, doctors spoke persuasively with the goal of recording a particular treatment recommendation  Conversations often incomplete when doctors/relatives disagreed about treatment recommendations; sometimes clincians said that information could only be delivered in stages and not all at the same time | **INFORMATION:** Often limited to CPR discussions. Occasionally mentioned ICU; More in depth with “palliative care conversations”. Some evidence that limited to CPR due to concerns about “information overload” or confusing the patient if the contents of discussions didn’t look directly relevant to them.  **INFLUENCE:** Evidence of persuasion in ReSPECT conversations. “CPR has a 20% chance of success in someone my age, but much lower in someone your age” [Site 4, C08J and C09J] …. “if the patient had CPR, he’d be “so debilitated” if he survived, “so I would suggest not to do it”. Likewise, after mentioning ICU, the consultant said that witnessing “these treatments would be painful for your family” (Site 5, C01C)”.  **IDENTIFICATION:** ReSPECT conversations usually were triggered when acute sudden clinical deterioration/immediate risk of dying or cardiac arrest or sometimes when a clear defined EoL diagnosis obtained; other trigger factors – organisational prompts or prompts from patient or family member. Prompts from family member often when they were dissatisfied with not having any interaction with medical team and not knowing what’s going on  **ORGANISATIONAL:** Organisational prompts sometimes used but not explored in this study | “CPR has a 20% chance of success in someone my age, but much lower in someone your age” [Site 4, C08J and C09J] …. “if the patient had CPR, he’d be “so debilitated” if he survived, “so I would suggest not to do it”. Likewise, after mentioning ICU, the consultant said that witnessing “these treatments would be painful for your family” (Site 5, C01C)”. |
| Griffiths et al 2020 | While doctors aspire to make patient-centred decisions, key challenges include: being overworked with lack of time; limited support from senior staff; and a lack of adequate staffing in other parts of the hospital that may be compromising patient safety. To reduce decision complexity, heuristic rules based on experience are often used to help think through the problem; for example, the patient’s functional status or clinical gestalt. The intensive care doctors actively managed relationships with referring doctors; acted as the hospital generalist for acutely ill patients; and brought calm to crisis situations. However, they frequently failed to elicit values and preferences from patients or family members. They were rarely explicit in balancing burdens and benefits of intensive care for patients | **INFORMATION:** Patients varied as to what they considered as a good QoL.  **INFLUENCE:** Patient can be too unwell to give a considered view on treatment options.  Patient and family have divergent views or family doesn’t want to talk about life-death decisions. | ICU registrar: “I had a patient...who said, “If I can walk. . .to the end of the bed I would think I have a good quality of life”. And another who’d say, “If I can’t go swimming every day I don’t think there’s any reason (to live)”  In the emergency department: The patient looks grey, having difficulty talking, out of breath, panic in their eyes, takes the oxygen mask off repeatedly, seems to be in agony. . .the junior ICU registrar asks the patient rapid questions about functional status, to which he says yes giving the impression he is in good health. . .the ICU registrar asks the wife and daughter outside the resuscitation room about the patient’s functional status and they give an a more pessimistic picture compared to the patient’s (field notes); In the emergency department: Patient seems uncomfortable, twisting in the bed. The CPAP machine is loud. Several staff around the bed doing procedures, such as blood sampling. The senior ICU registrar asks in a loud voice: “Have you thought about what would happen if you get worse?” [No audible answer] “Do you want dialysis?” The patient says “yes”. . .“Would you want to be put to sleep on a breathing machine?” Patient rotates her hands as it trying to say she doesn’t know. Field notes 4:2  Patient:” I have said. . .if I was really ill, a burden, haven’t got anything going for me, I would rather not (go to ICU). . .but my son says don’t talk so silly. . .he doesn’t like talking about things like that” |
| Haliko et al 2018 | 10% provided patient-discordant treatment and intubated the patient.  Physician reluctance to disclose uncertainty to patients and patients reluctance to communicate physician error were risk factors for patient discordant treatment | **SKILLS:**  Poor insight into EoL situation: Lack of skills responsible for discordant treatment. This includes general lack of awareness of the importance of patients cultural values and what’s important to them. This leads to focussing on the medical problem and not the overall picture. Intubators (discordant) more often described the patient situation using terms related to “emergency”, “reversibility” and “imminence” relating to intuitive rather than analytical decision making process.  Poor recognition of true autonomy  Intubators emphasized their own perceptions of the case more than non-intubators. When intubators did discuss patient preferences, they more frequently mentioned “inferred” over “explicitly stated” patient preferences  Lack of confidence: Discomfort with case was mentioned in the context of increased caution (i.e. uncomfortable bringing up EoL situation with patients). | “if I intubate him today and let us say that he has pneumonia or he is septic... a couple of days of antibiotics can reverse all of that stuff and he can come back  “[I] basically said in very small increments ‘I am escalating care’, and would look at him and the family member [implying the question] ‘is this okay with you?’... It felt like I had no resistance ...towards aggressive care, so I continued to get more aggressive and escalate care.”  “It is always difficult to approach...It’s not always difficult, well, it is not always difficult, but it’s a bit uncomfortable approaching that particularly in the presence of the patient, hence the hesitation.” |
| Harris et al 2021 | Themes:  Preparedness and unpreparedness to have GoC discussion. Some perceived the GOC discussion as irrelevant or inappropriate often related to their perception of good health. The beneficial influence of previous GOC discussion experience on readiness was highlighted by doctors and patients alike  Challenging logistics   - Distraction and disorientation during GOC discussions (CMO7) - No ideal time for GOC discussion (CMO2) - Importance of family presence (CMO2)   Communication and understanding   - GOC communication is focused on life-saving procedures - Difficulty understanding doctors - GOC is a discussion about death (CMO2) - Uncertainty about doctor understanding of patient values   Multiparty decision making   - reliance on clinical expertise in GOC decision-making (CMO3) - Influence of family on GOC decision-making (CMO2) | **INFORMATION**:  Preparedness to have conversation. Are patients prepared to have such conversations? If not, some might be perceived as inappropriate  And beneficial impact of having previous experience of GoC or EOLC conversation  GOC is a discussion about life-saving procedures and death.  Uncertainty about doctor understanding of patient values  Conversations are often procedure focussed with limited understanding of patient/family  A few patients strongly emphasised the importance of listening, supporting a two-way discussion over an information giving session. Some felt fast speech hampered understanding and others reported difficulty understanding content  **INFLUENCE:**  Disorientation from the effects of acute illness and disordered hospital environment.  From patient perspective, importance of family presence  But doctors did not always feel family presence was needed.  Doctors actively looking to influence patient decision-making:  **RELATIONSHIP:** Patients, family and next-of-kin consistently described doctors as caring and efficient and trusted in their aim to support the patient’s best interests. Patients rely on clinical expertise, which in  **IDENTIFICATION:** Some perceived the GOC discussion as irrelevant or inappropriate often related to their perception of good health  **ORGANISATIONAL:** Distraction and disorientation during GOC discussions. No ideal time for GOC discussion | “...I don’t think I was bad enough...health wise...to warrant that sort of situaiton...I thought no, but I went along with it because you do that when you are in hospital. You go along with things.”  “It’s been constantly thought about since, ah, the last event.”  “Well, they are asking whether I would like to have everything turned off. Or I want to be resuscitated.” (Patent 8) “End of life, ah um what treatment is ah given or expected prior to the end of life.” (Next-of-kin 1) “...I don’t like the idea of associating people coming to the hospital with having a discussion about their mortality...I think it's not part of life...death is the end of life...life is about living it...it really sort of plants unpleasant seeds, unnecessary seeds of the end of life.” (Patient 2)  “I don’t know. They always seem to be different.” (Pa􏰀ent 10)  “...she understood what resuscitation meant... but she didn’t clearly understand what intubation and non-invasive ventilation meant... What do those mean in context?” (Doctor 3)  “...you are disoriented enough without, you know, getting involved in everything else.”  “I think it’s a joint decision... I would like to do it in conjunction with my kids.” (Patient 6) “In that situation I think I’d rather have my family, I really do. I know it might be upsetting for them but I s􏰀ll would like them to be involved.” (Patient 8).  “She was competent enough to make the decision... I didn’t feel that a family member needed to be there or needed to be contacted because [the patient] can make her own decisions.”  “...probably the big influence was the way I sold it to him... sometimes I’ll push my agenda more obviously if I disagree with them to try to get them to my same page... it’s [medical intervention] not the best thing for people sometimes, but they think it is.” (Doctor 2)  “Because somebody has explained to you, you know, that your heart has changed shape, I didn’t know that because it’s not my area of expertise....And you have to trust somebody who’s got more knowledge than you to make those decisions on your behalf.” (Patient 6) |
| Hart et al 2021 | Physicians correctly answered 4.85 correct out of 8 choice architecture competency items (SD=1.26; 95% CI 4.59 to 5.11)  Physicians demonstrated the highest competency in scenarios assessing habit formation, social norms and default effect. They demonstrated the lowest competency on scenarios assessing relative risk bias, anchoring effect and multiple alternatives bias  Qualitative interview themes  Choice architecture is highly relevant to healthcare  Physicians predict influences based on personal experiences  They viewed the intentional use of choice architecture as acceptable and ethical, but felt they lacked sufficient formal training in the principles to do so. Most “learn on the fly” and by trial and error  Ethical boundaries exist when using choice presentation and often influence patients choices/autonomy | **INFLUENCE:**  Clinicians have awareness that they often try to influence patient choices:  Biases that can influence clinicians communication with families. Brackets represent percentage of clinicians were competent/aware of such biases: Anchoring bias (29%): people tend to rely on the first piece of information or “anchor” when making decisions; Compromise effect (66.7%): A less attractive or less preferable choice increases the attractiveness or another, alternative choice; Default effect (81.7%): the default effect is the result when no explicit decision for an alternative option is made; Framing effect (62.4%): People tend to prefer certainty in a gain frame (ie, saving lives) and uncertainty in a loss frame (ie, losing lives); Habit formation (88.2%): People tend to adhere to their routine activities and resolutions if they engage them on a regular basis; Multiple alternatives bias (14%): A multiplicity of options may lead to significant conflict and uncertainty, resulting in lack of a decision or a response of ‘I don’t know’ or ‘let me get another opinion’; Relative risk bias (57%): People tend to interpret relative risk differently when presented as percentages and when presented as ratios. People often interpret ratios more strongly than percentages.; Social norms (86%): Rules or behaviours that are considered socially acceptable by a cohort or group. It is understood by all members of the group that they should abide by these norms.  **SKILLS/CONFIDENCE:** No specific choice architecture training | “Depending on how you’re presenting [choice options], you’re going to influence patients’ decisions. But that’s kind of your job in the role of an expert consultant, is to influence or recommend. I think most people when they are influencing peoples’ decisions are doing it from the place of trying to do what they think is best for the patient. I think that is always ethical...”  “I have learned these [decision-making principles], but not in such an explicit way. It’s more just through experiencing how my attending[s] talk.” |
| Hayes et al 2010 | Qualitative. Grounded theory Declining Trust in medical profession over time  Trust is a pre-requiste to have CPR discussions. Patients need to be able to trust that the doctor having discussion with them knows the likely outcomes in this patients case (i.e. has sound medical knowledge and technically competent).  CPR discussion itself may erode the patient or families trust in the moral character of the doctor (e.g. not for resuscitation were sometimes interpreted by patients as having hidden meanings - e.g. doctor not wanting to try as hard or wanting to end the patients life/giving up on them  The effect of cultural values on Trust. Some cultures do not believe in NFR and expect CPR to be done regardless and if not, this can dissolve trust  CPR discussion itself has the potential to undermine trust and create distrust if the patient comes to this discussion with an ethical view about CPR that is not shared or recognised by the clinician  Patient’s capacity to trust  e.g. previous bad experience leading to loss of trust in doctors generally  Building Trust   - Addressing lack of trust directly with family - Spending time with family to build rapport and listening to understand person and values (can use nurse as facilitator in this process) | **INFLUENCE**: television/internet reporting of distorting patients and families views of outcome relating to CPR.  **RELATIONSHIP:**  Declining Trust in medical profession over time  Trust is a pre-requiste to have CPR discussions. Patients need to be able to trust that the doctor having discussion with them knows the likely outcomes in this patients case (i.e. has sound medical knowledge and technically competent  CPR discussion itself may erode the patient or families trust by interpreting that the doctor won’t do their all for them  The effect of cultural values on Trust. Some cultures do not believe in NFR and expect CPR to be done regardless and if not, this can dissolve trust    CPR discussion itself has the potential to undermine trust and create distrust if the patient comes to this discussion with an ethical view about CPR that is not shared or recognised by the clinician  Patient’s capacity to trust e.g. previous bad experience leading to loss of trust in doctors generally  Building Trust: Addressing lack of trust directly with family and spending time with family to build rapport and listening to understand person and values (can use nurse as facilitator in this process)  **ORGANISATIONAL**: The effects of “NFR” on how healthcare professionals treat/manage/prioritise their patients | “Television shows...they see all this magical stuff done, and when it comes to their loved one... they think there should be no reason why this magical stuff shouldn’t be able to be done. I think there are often unreasonable expectations” (Senior doctor 4). “No longer does it take 6 years basic training as a doctor and specialist training for years after to make that decision, to be armed with the information. Whereas they can look it up in five minutes on the internet, the condition, and become instant experts...and I’ve had the conversation...and it’s almost like they’re waiting, they’re trying to trip you up” (Nurse 4)  “I feel day by day there is lack of trust...they are cautious...you don’t feel that trust between you and them...you have to prove what you say on everything” (Senior doctor 8)  ”Well I think it’s a huge thing because they’re well...especially if you’re telling the patient and their family what you think is the right or wrong thing, there’s a huge element of trust...they’re trusting that you know enough about their illness to know what the likely prognosis and what the likely survival is (Senior doctor 11).”  “They have a misconception. They think if you have one of those orders filled in then the doctors don’t try as hard to treat them; they won’t give them all the other medical treatment that they need (Junior doctor 2).”  “In the Middle East you do things basically until even after death. There’s no way you can stop and say—I will not resuscitate or I will not do this or there’s nothing more... People coming to western countries from the Middle East would be quite not accepting of the idea of stopping treatment, their idea is to have treatment... In the Middle East you would never discuss with the patient resuscitation or decision-making (Senior doctor 8)”  “The aim there is really to try and build rapport...so they end up trusting...particularly if they see us continuing the treatment…”  “I think there’s a lot of misunderstandings, both medically with junior medical staff and others often—if you have a NFR order, that does not mean that you don’t do everything [else] (Senior doctor 2).” |
| Hutchison et al 2016 | 5 themes of trust identified:  Technical competence  (relating more to nursing staff)  Communication: use of lay language, frequency of updates  Honesty: receiving accurate, truthful information  Benevolence  Interpersonal skills: introducing themselves when entering a room, addressing surrogate by their name/remembering their name; caring/personable and warm  On a hospital/healthcare system scale – trust based on feedback from relatives/friends who have received care there, hospital reputation and prior personal experiences | **RELATIONSHIP/SKILLS:** 5 dimensions of Trust:  technical competence: nurses diligence, teamwork and confidence were relevant behaviours affecting trust. Comments regarding doctors infrequently discussed    Communication: use of lay language, frequency of updates  iii) Honesty: receiving accurate, truthful information  iv) Showing compassion    v) Interpersonal skills: introducing themselves when entering a room, addressing surrogate by their name/remembering their name caring/personable and warm  **ORGANISATIONAL**: Trust in the hospital and healthcare system improves overall trust. Due to endorsements by family members or friends, reputation or prior personal experiences at the medical centre. “Some felt that having a relationship with an outpatient physician at the medical center, such as an oncologist, provided a sense of comfort and augmented trust in the ICU clinicians” | “And then the nurse X who would stay on all day just was extraordinary. She never stopped doing things. I mean, she was on her feet all day adjusting, titrating, you know, getting things the way they should. It was really very, very impressive...And I think another good experience was how quickly from when the bell went off, if a number was askew, they were right there.”  “They explain it to me in a way that I can understand. They gain my trust because they’re not leaving pieces behind. I’m understanding what they’re telling me. I think that will help you gain trust in a person.”  “So to me that’s what trust meant, that this is valid information, it’s not being shaded, it’s not being manipulated, this is the real story. So that’s what it means to me.”  Every day there is somebody who walks in with sepsis, but for the person who has it and for the people who are around them it is not real. So that is what I would say: sincerity and compassion go a long way in building trust because once you have us, once we believe in you then we will listen to you.”  “And he gave me that glimpse of his personal life, which, I thought, ‘Ahh! You do understand. You’re not just in a white coat and not a human being. But you get it’... They gave you that little insight of their personal life...I appreciate that.” |
| Kon et al 2016 | 6 recommendations: 1) Definition: Shared decision-making is a collaborative process that allows patients, or their surrogates, and clinicians to make health care decisions together, taking into account the best scientific evidence available, as well as the patient’s values, goals, and preferences. 2) Clinicians should engage in a SDM process to define overall goals of care (including decisions regarding limiting or withdrawing life-prolonging interventions) and when making major treatment decisions that may be affected by personal values, goals, and preferences. 3) Clinicians should use as their “default” approach a SDM process that includes three main elements: information exchange, deliberation, and making a treatment decision. 4) A wide range of decision-making approaches are ethically supportable including patient- or surrogate-directed and clinician-directed models. Clinicians should tailor the decision- making process based on the preferences of the patient or surrogate. 5) Clinicians should be trained in communication skills. 6) Research is needed to evaluate decision-making strategies. | **INFORMATION** Default SDM strategy is recommended by the authors of this paper that includes 3 main elements: information exchange, deliberation and making a treatment decision. Clinicians share information about the relevant treatment options and their risks and benefits, including the option of palliative care without life-prolonging interventions, and the patient or surrogate shares information about the patient’s values, goals, and preferences that are relevant to the decision at hand. Next, both clinicians and patient/surrogate share in deliberations about which option is most appropriate for the patient. Finally, clinicians and patients/surrogates agree on the decision to implement; further research re decision aids  **SKILLS/CONFIDENCE**: Clinicians should be trained in the communication skills necessary to create effective partnerships in treatment decisions. Core categories of skills include: 1) establishing a trusting relationship with the patient/surrogate; 2) providing emotional support; 3) assessing patients’/surrogates’ understanding of the situation; 4) explaining the patient’s condition and prognosis; 5) highlighting that there are options to choose from; 6) explaining principles of surrogate decision-making; 7) explaining treatment options; 8) eliciting patient’s values, goals, and preferences; 9) deliberating together; and 10) making a decision; Further research required regarding communication skills training  **ORGANISATIONAL:** Further research re use of patient navigators or decision support counsellors |  |
| Kryworuchko et al 2016 | The overarching factor was how people conceptualised the goals of medical practice as saving lives/avoiding death unless death imminent (therefore discussions often delayed/avoided) until clearer that LST was futile  Communication with patients/families often stressful with focus on “getting the DNR”  Work towards “making sense of the situation” (establishing relationship with pt/family, integrating info about patient’s health, treatment options, illness belief and goals of care. Prognostic uncertainty often resulted in HCPs not making sense of the situation  Family emotion can lead to irrational thoughts (or being “difficult” about management and increased tension between them and HCP. when communications with families did not go well, most nurses and physicians did not have the experience and skill set to unblock tense disagreements, nor did they have much time to do so  Leading emotional end-of-life discussions alone placed a great deal of pressure on physicians. work done by nurses was not recognised as contributing to communication and decision-making about life-sustaining technology (even for nurses themselves). Nurse participants described their work as hap- pening asynchronously around physicians’ encounters. They described preparing the groundwork for physicians’ discussions about life-sustaining technology, as well as ‘picking up the pieces’ after physicians delivered unexpected prognostic news and decisions  Residents recognised importance of more training. Mentorship, modelling and support to develop the necessary skills to engage patients and families in productive discussions were frequently raised by participants | **INFORMATION**: To order to provide personalised information, HCP needs to understand patients own thoughts about their health, treatment options, illness belief and goals of care.  **INFLUENCE:** Family emotion can lead to irrational thoughts (or being “difficult” about management and increased tension between them and HCP.  **RELATIONSHIP:** Establishing a relationship with family important to open up a 2 way dialogue which is important for HCP to get to know the patient  Wider societal view of death as a medical failure and unaddressed fear of “abandonment” can break down trust and good relations/mutual understandings.  Trust is lost in situations when communications didn’t go well  **SKILLS:** Communication with patients/families often difficult and are fearful in EoL discussions. Leading emotional end-of-life discussions alone placed a great deal of pressure on physicians.  HCPs find prognostic uncertainty challenging and not being able to “make sense of the situation”.  When conversations didn’t go well, most HCPs did not have the experience and skill set to unblock tense disagreements  Further training welcomed by HCPs along with mentorship, modelling and support  **IDENTIFICATION:** Discussions avoided until life-saving was not possible or death occurred.  **ORGANISATIONAL**: HCPs did not have much time to unblock tense disagreements in situations when HCPs and patient/family have discordant views in clinical management.  Nurses view their role as preparing the groundwork for physicians discussions about life sustaining technology, helping patients and families make sense of the situation and “picking up the pieces” after physicians delivered unexpected prognostic news and decisions.  Nurses may feel inhibited from influencing physicians decision about patient management. | ‘We know everything medically about them, but we don’t know their story and we don’t know what informs the decisions they’ve made to this point” (Resident Physician)  ‘It’s easy when everyone is thinking the same thing.’ (Staff physician  ‘One of the daughters was angry. She was saying we were abandoning her mother. That we weren’t allowed to do that. That we had to keep it up until the end.’ (Nurse)  “sometimes it’s about us, we’re not comfortable making that decision either” (resident physician) and ‘And I can say this with certainty, that there are people, and I’ve seen it with colleagues as well as students, who are afraid of this: who are afraid of talking about anything related to end of life with people.’ (Staff physician  ‘A lot of times when the physician is having that conversation on a medical unit, it’s when things have gone badly, when things have changed, when the patient is doing poorly so the family is really distressed about how their family member is doing.’ (Nurse)  ‘Sometimes it’s denial; sometimes it’s that we don’t have time” (resident physician)  They’ve just been told something potentially devastating. So you’ve got to ask how much they actually retained. So that’s usually the best place. So gleaning a bit of an insight into what they understand, what they retain, what this means to them or what they’re understanding it means, is probably the biggest step for the nurse to take after they’ve had that change.’ (Nurse)  ‘It’s out of my hands whether or not it’s taken into consideration or not. You can tell residents all they want but if they have something set in their mind that this is going to happen then that’s going to happen. Most of the times we can’t change their minds. But you never know.’ (Nurse) |
| Lagrotteria et al 2021 | Qualitative semi structured 3 themes relating to SICP (perceptions of HCPs rather than data to show efficacy in each of the themes):  Supported changes in clinician behaviour  Supported by unit champion, improved interprofessional engagement, access to copies of the SIC guide and documentation in electronic medical record  Shifts GoC conversation – changing what is asked and creating space for conversations, facilitating understanding of patients illness and altering clinician agendas 🡪 conversations extending beyond code status  Influenced clinicians personally and professionally (increased comfort with having serious illness conversations, bringing meaning to their work and reducing moral distress and humanising care and tailoring care plan to support patients wishes.  Concerns with programme: finding time to have conversations, building transient relationships and limitation of conversation fluidity | **INFORMATION**: SICP facilitates families understanding of patient’s illness  **RELATIONSHIP:** being able to build a relationship in an busy acute environment    **SKILLS:** SICP increased clinicians comfort in having such discussions  Some perceived that the SICP inhibited the natural flow of conversation  Facilitated the “humanizing of care”. One physician described providing “a different type of care and really a kinder type of care,” and another clinician described having “a little more of an empathetic edge,”    **IDENTIFICATION:** SICP perceived to be able to identify patients who are likely to benefit from conversations earlier.  **VALUE**: SICP generally well accepted with clinician “buy-in”  Bringing meaning to work and reducing moral distress;  Having a conversation about serious illness and “getting the patient’s input into what we were doing” was described by another clinician as relieving a “burden.”  **ORGANISATIONAL**: Having a unit champion regarded as one of the most important elements  Documentation improves interprofessional communication: | “I had 1 family where there were things that [the patient] expressed that they hadn’t known...it was clear that this family hadn’t gone through this before, or the kids had not appreciated their father’s concerns...they came later and really thanked me for having [the conversation].”  “I think what I found most useful was a separation of time and space; [it] created a moment to build a relationship in a way that we don’t always in acute care because we don’t either have the time or the dynamic is different.”; Getting to know what’s important to the patient: “[The guide] creates a completely different environment because you’re asking questions about their bigger life values and goals...We don’t ask these things. It’s really pivotal for me; it really changes my practice.”  “…I might not have asked some of those questions before, but I’m far more confident to do it now.”  “I just think just the static nature of it makes it a bit difficult to adapt to an actual, real- life conversation sometimes.”  “when it’s so busy, it’s easy to...and I don’t use it as a bad term in a bad way...dehumanize people and process people. Just because you’re trying to get through, you become mechanical in what you say and what you do.”  “I think we’re doing a better job of identifying these patients earlier, and whether it’s attributable to this program, specifically, or this is just supporting a culture shift in that regard, I’m not very sure.”  “I also feel like the whole team has embraced this process from all the varied allied health professions. Everyone is very aware of the process and so, when someone brings it up, it’s like, ‘Yeah, you’re right. That would be appropriate.’ I think we’re all so invested in this process because we know it helps and it helps to give better care to our patients.” ; “I realized how patients are so willing to open up and speak, and it actually does work, some of these specific questions, of areas to explore. It allows me to be more using of it, and it’s nice using something that’s been validated, researched, so it really adds to the toolbox”  “It definitely...makes the day feel much more fulfilling to be able to connect with the patient or family on that deeper level as opposed to the more superficial and very busy tasks of the day.  “There’s a cueing reminder, there’s an administrative burden removed where someone else is scheduling and letting me know when it [the conversation] is, based on my availability, and that really helps. The patients and family are primed on it. It was just any time I wanted to do it, and whenever we planned to do it, it just happened. Everyone was on board, and everything was set up.”; Having copies of the guide accessible “The sheets with the conversation guide that you can take off the wall and take into the patient's room with you. You can just take it and do it, like, right there. So, that makes it really easy to use.”  “If you're dictating in a conversation, it’s clear to everyone that this is what this person wanted, and it’s dictated, it’s easy to pull up, people readily access it. Even the dictations that I’ve dictated...I have had people readmit and they get readmitted to our team and even it’s been some time later. It’s like, okay, this is what the person wanted in this serious illness conversation at this time.” |
| Loewenstein 2005 | When patients are in “cold states” they may fail to fully appreciate how hot states will affect their own preferences and behaviour.  When in hot states, they underestimate the influence of those states and overestimate the stability of their current preferences. The same biases apply inter-personally (people who are not affectively aroused underappreciate the impact of hot states on other peoples behaviour).  In medical decision making, hot to cold empathy gaps (brought by stress, anxiety, emotion) cause people to underestimate the extent to which these states are influencing their bahviour and tend to overestimate how long they will continue to experience their current feelings and preferences. This could provide a rational explanation as to why patients/families often change their minds wishes and preferences | **INFLUENCE:** The timing of having conversations about LSTs relates to the degree of hot or cold emotional states that the patient and/or family experiences in relation to illness behaviour (including articulating wishes for ongoing or future treatment). This may limit the usefulness of ACP (when in cold emotional states) and account for less rational/irrational thought processes in hot states. The impact of how emotional states in relation to LST is beyond the focus of this review but it’s influence on medical decision making needs to be accounted for. |  |
| Lee et al 2022 | Jumpstart intervention was associated with more documented GoC conversatioins (16  of 75 patients [21%] vs 6 of 75 patients [8%]; risk difference, 13%[95%CI, 2%-24%]; risk ratio, 2.67  [95%CI, 1.10-6.44]; P = .04)  Patient-reported or surrogate-reported goals-of-care discussions did  not differ significantly between groups (30 of 66 patients [45%] vs 36 of 66 patients [55%]),  No significant difference with respect to patient reported quality of communication  Acceptability equivocal  Intervention appeared feasible | **IDENTIFICATION AND ORGANISATIONAL:** The Jumpstart guide is a GoC conversation primer which appears feasible and acceptable and possibly effective at increasing GoC conversations in a small pilot study that includes 150 patients. However, this trial’s main bias is potential contamination of controls from the intervention arm due to the primer being on the same study site. Also, the intervention is resource heavy (research staff hand-delivered questionnaire and conversation priming sheets to both patients/surrogates and medical staff). Its internal and external validity needs to be further tested in larger research trials (probably cluster RCT design). |  |
| Levinson et al 2019 | 19 themes originally condensed to 5 themes after consensus.  Mixed understanding of “palliative care” and “GoC” terminology  ED doctors focus on short term goals of care (particularly on acute medical therapies) and not necessarily the longer-term goals of care in mind specific to the patient  ED doctors delt that GoC discussions were relevant to their practice and often necessary  Using positive language to describe treatment limitations (i.e. discuss the care that will be provided rather than the care that will be held)  Training requirements for doctors felt important (workshops, clinical experience, guidelines)  . | **INFORMATION:** Different approaches from those who have and haven’t been identified as those who are at the EoL. For those not at EoL, Doctors tended to focus on the acute medical therapies they would provide with little emphasis on patient preferences, existential issues, or longer-term goals-of-care.  Those who are known EoL, there is a different approach  **RELATIONSHIP**: ED physicians felt they were disadvantaged when holding goals-of-care discussions because they had to establish a trust under difficult circumstances and often did not know the medical history beforehand  **SKILLS:** Many ED doctors thought it was important to discuss the care that would be provided, rather than what would be withheld.  Senior ED doctors believed that junior doctors required training and mentoring to be successful in this task. Most suggested that junior staff needed to attend workshops or simulations and accompany senior doctors to gain practical experience and learn to manage different scenarios. A few desired guidelines.  **VALUE**: The intent of GoC dicsussions appeared to prevent non-beneficial treatment during acute hospitalisations prior to medical or surgical review on the ward. | “Maybe we’re guilty. . . of having preconceived goals-of-care in our own mind from a medical perspective, rather than actually sitting down with the patient’s family and making sure they have similar sense.”  “The palliative care patients. . . often those conversations are around just establishing what the patient understands of their treatment, and what treatments are acceptable to them.”  “Our disadvantage is. . . those patients who are seeing us for the first time, families who haven’t met us before we don’t have that established therapeutic relationship so that trust isn’t always there and we have to work very hard to establish that trust.”  ‘The two things that always come up is are you withdrawing treatment and are you giving my relative tonnes of morphine to kill them. . . this is why sometimes specifics help. We talk about fluids, feeding, antibiotics and sedatives.  ‘If. . . the patient is in a critical situation, yes we should do it in emergency, because otherwise maybe he (sic) will get treatment that is inappropriate on ward.’ |
| Lindberg et al 2015 | Patients in ICU wanted to be involved in decisions about their care as this creates a trusting and healthy environment  Patient autonomy was shown to be a “trajectory towards partnership”:  Acknowledged dependence when sick (feeling trust, surrendering, losing control, accepting dependence)  When recovering:  Being recognised as a person (being noticed, being asked, being listened to, being shown respect, being given information  Invited participation (being encouraged, being invited to initiate care activities, being part of the care relationship) Becoming a co-partner in care (participation in decision making, exerting an active influence on care, experiencing independence, taking personal responsibility) | **RELATIONSHIP:** i) Acknowledged dependence when sick (feeling trust, surrendering, losing control, accepting dependence)  When recovering: ii) Being recognised as a person, iii) Invited participation (being encouraged, being invited to initiate care activities, being part of the care relationship) Becoming a co-partner in care (participation in decision making, exerting an active influence on care, experiencing independence, taking personal responsibility). | ‘‘... in the ICU ... it was never an issue for me to have views on (care decisions) . . . I realised that they (the staff) were doing things that were necessary.’’ |
| Lu et al 2015 | 5 frames were identified: will (decided), must (necessary), should (convention), could (option) and ask (elicitation)  Physicians broached LST differently than palliation (p<0.01) most commonly framing LST as necessary (must) (53%), while framing palliation as optional (could) (49%)  Among physicians who framed LST as imperative (will or must), 16 (30%) felt intubation would be inappropriate in this clinical situation | **INFORMATION:** With respect to options on management, intubation was discussed more frequently than palliation: 64% broached intubation but did not broach palliation until surrogate expressed preference to avoid intubation. 12% didn’t broach palliation at all; 8% broached both treatment options  **INFLUENCE**: 5 frames were identified: will (decided), must (necessary), should (convention), could (option) and ask (elicitation). Physicians broached LST differently than palliation (p<0.01) most commonly framing LST as necessary (must) (53%), while framing palliation as optional (could) (49%). Also, examples where intubation has been “normalised”  Among physicians who framed LST as imperative (will or must), 16 (30%) felt intubation would be inappropriate in this clinical situation | “What we **normally** do for cases like this is we put people on breathing machine and you know what that entails, sir?” |
| Mentzelopoulos et al 2021 | 5 top plans with themes relating to advanced care plans, educating patients and the public, educating healthcare professionals, when to start and stop resuscitation and research  Healthcare systems should provide clinicians with communication skills training interventions to improve clinicians’ skill and comfort (CMO 7)  Clinicians should integrate the following patient/family support elements with shared decision making: 1. Provide information about the patient's status and prognosis in a clear and honest manner. This may be supported by use of a video-support tool. 2. Seek information about the patient's goals, values, and treatment preferences. 3. Involve patients/family members in discussions about advance care plans. 4. Provide empathic statements assuring non-abandonment, symptom control, and decision-making support. 5. Provide the option of spiritual support. 6. Where appropriate, explain and apply protocolised patient centred procedures for treatment withdrawal with concurrent symptom control and patient/family psychological support. 7. Consider recording meetings with family for the purpose of audit/quality improvement.  Other relevant messages (based on the context of ACP rather than acute hospital setting):  Patient and family satisfaction is a key objective of patient- and family centred communication and care. Key components of this approach include open, honest, clear, and frequent communication and inclusion of family members in discussions with healthcare professionals. The use of structured communication tools may help to improve communication with families. Communication supported by other strategies such as video decision aids may be associated with improved family satisfaction | **INFORMATION:** Clinicians should integrate the following patient/family support elements with shared decision making: 1. Provide information about the patient's status and prognosis in a clear and honest manner. This may be supported by use of a video-support tool. 2. Seek information about the patient's goals, values, and treatment preferences. 3. Involve patients/family members in discussions about advance care plans. Provide empathic statements assuring non-abandonment, symptom control, and decision-making support. 5. Provide the option of spiritual support. 6. Where appropriate, explain and apply protocolised patient centred procedures for treatment withdrawal with concurrent symptom control and patient/family psychological support. 7. Consider recording meetings with family for the purpose of audit/quality improvement.  **TRUST/RELATIONSHIP:** Patient and family satisfaction is a key objective of patient- and family centred communication and care. Key components of this approach include open, honest, clear, and frequent communication and inclusion of family members in discussions with healthcare professionals  **SKILLS/CONFIDENCE:** Healthcare systems should provide clinicians with communication skills training interventions to improve clinicians’ skill and comfort. The use of structured communication tools may help to improve communication with families. Communication supported by other strategies such as video decision aids may be associated with improved family satisfaction |  |
| Periyakoil et al 2015 | 99.99% doctors reported barriers with 85.7% finding it very challenging to conduct EOL conversations with all patients and especially so  with patients whose ethnicity was different than their own.  Largest physician reported barriers:  Language barrier and medical interpretation  patient/family religio-spiritual beliefs about death and dying  Doctors ignorance of patients’ cultural beliefs, values and practices  patient/family's cultural differences in truth handling and decision making  patients’ limited health literacy  patients’ mistrust of doctors and the health care system  The doctors' ethnicity and medical subspecialty (Chi-Square = 19.33, DF = 10, p =0.036) influenced  their reported barriers | **INFORMATION**: Patient/family’s limited health literacy  **INFLUENCE:** Language barrier and medical interpretation  Patient/family religio-spiritual beliefs about death and dying  **TRUST/RELATIONSHIP:**    Doctors ignorance of patients’ cultural beliefs, values and practices patient/family's cultural differences in truth handling and decision making  **ORGANISATIONAL:** patients’ mistrust of doctors and the health care system | “Certain medical terms may be difficult to explain in a way the patient can understand.” “They may not be used to the health system they find themselves in and it may be overlooked that they lack what we would consider common knowledge” “Incomplete understanding of what resources/therapies that can be versus should be provided for a patient.” “Misunderstanding what is described by resuscitation, thinking it means we are giving up completely on treatment”  “Difficulty in translation, sometimes interpreters may not exactly translate the feeling and meaning of a conversation.”  “Religious cultural values that may lead people to prefer life-sustaining treatments that we may see as futile.”; “Hoping for religious miracle.”; “Reconciling religious "obstructions" to a Do Not Resuscitate status.”  “Connecting emotionally to the patient and/or family through an interpreter (is a barrier)”  “Doctors not understanding the cultural values surrounding end-of-life care for a patient with a different ethnic/religious background.”; “Unfamiliar with social norms for showing sympathy, hug? cry?”; “Cultural norms that differ from my own causing me to inadvertently offend the patient or his/her family.”; “Not understanding which topics might be taboo.”; “Not knowing how to discuss goals in a way that makes sense to someone with different views about death based on different beliefs about spirituality and afterlife.”;  “Certain groups feel that honesty and frankness is not good for the emotional outlook on life for the patient and they do not want the physician to be frank with the patient in discussing end-of-life issues at all.” “I think it's fair to say that some cultures approach (conversations about) death as something to be avoided at all costs, which is not necessarily how i, as a health care provider, feel about it.” “In some cultures (i.e. Asians), patients may not want their diagnoses/prognoses discussed with them directly and will instead appoint a family member as surrogate decision maker. It can become difficult however, to be sure that that family member is acting in the best interest of the patient and acting with the patient's preferences in mind vs. their own.” “Different opinions on the role of patient autonomy in making end-of-life decisions understanding that in some cultures the decision making may fall to a different member of the family than the patient.” “Eliciting the personal wishes of a female from a culture in which men make all the decisions can be difficult.”  “Some groups feel more marginalized in the community at large and this makes them more distrustful of the medical system as a whole.”; “Patients may believe that care is being "withdrawn" from their loved one because of racism.” “Certain cultures lack trust in the medical profession, do not believe physicians have their best interests at heart.” “Fears of abandonment or self-interested medical professionals”. |
| Pham et al 2008 | An alteration during 55% of all interpreted speech passages (322/583)  Alterations included additions, omissions, substitutions and editorialisations  Over 3 quarts of alterations were judged to have a potentially clinically significant consequences on the goals of the conference (defined in table 3).  93% likely to have a negative effect on communication (including interference with transfer of information, reduced emotional support and reduced rapport); the remainder a positive effect (improvements in conveying information and emotional support) | **INFLUENCE:** alterations during passages of speech either by clinician or patient/family in the presence of a language interpreter were common (55% of all interpreted speech passages Over three-quarters of alterations were judged to have potentially clinically significant consequences on the goals of the conference.  **RELATIONSHIP:** Could have a negative implications relating to rapport and emotional support, which are discussed in paper but no examples in extracts relating to this | Extracts: “**MD** I don’t know. Um, this is a very rapidly progressing cancer. **Interpreter (translating)** He doesn’t know because it starts gradually; “**MD** The problem with this option is that he may have to stay on this machine for the rest of his life. **Interpreter (translating)** But the problem with this option is that he will have to stay on this machine for the rest of his life”  **Family** But, what we want to know is that after his lungs get better and when he wakes up will his brain suffer and affect his ability to recognize people? **Interpreter (translating)** Okay, she wants to know about the lungs, when he wakes up, so about his lungs, and so, what about after, so it will not affect him? **MD:** but my best hope is that his lungs would be as good as they were before. But they won’t be better than they were before”. |
| Pollack et al 2019 | No difference in the presence of documented GOC conversations. In the intervention arm, 49% included a description of a GOC conversation compared with 50% in the control arm (OR 0.97; 95% CI 0.64 - 1.46; P=0.8616)  Quality of communication (defined by SUPER) was improved on 2^nd^ 30 min coaching session compared to first.  S: set-up environment  U: understand what pt/families know  P: prognosis/priorities  E: emotion – expect, recognise, respond  Recommend/review  Intervention acceptable and was well received ‘‘The personalized assessment and re- view of my encounters with patients was most helpful. I learned what I was doing well and was given insight as to why the various techniques were effective.’’  Several limitations:   - Multiple biases including potential contamination from intervention to control arm at single study site - V small sample sizes   🡪 further research required | **SKILLS:** Coaching intervention designed to improve GoC communication with patients was well received by clinicians. In this study it involved 1 hour teaching initially, followed by 2x 30 mins coaching on recorded transcripts and feedback where appropriate.  The 2^nd^ feedback session was suggestive of this coaching meeting its intended aim (in this case compliance with SUPER algorithm), albeit pilot study and not definitive.  **IDENTIFICATION:** Electronic health alerts based on pre-defined criteria (combined with coaching intervention) did not improve the number of documented GoC conversations. |  |
| Rassmussen et al 2018 | Five influencing themes were identified: duty to defend (duty to care for loved one); been here before (influence of past experiences); severity of situation (impact of critical ill- ness requiring ICU admission); ACP is power (prior engagement helped decrease stress); and true trust of HCP). | **INFORMATION:** avoiding medical jargon  **INFLUENCE:** Differing views from different family members  Influence of prior experience gives greater insight  Acute illness impacts any meaningful conversation with patients, sometimes with little time to have conversations due to urgency of situation. Also the situation causes lots of emotion and “shock” which might impede any meaningful conversation. Therefore, talking ahead of time helpful  **RELATIONSHIP/TRUST:** Openness and compassion viewed positively | “…and he made complete sense to me... he talked in enough layman’s terms that we could understand... the issues that he was describing”  “I really struggled at the start of all this with some family input, and it was like “You can’t make that decision, you don’t know what he would want!” You know, so I found that really hard. And it was like: “I live with him, I know what he wants.” And you know, I get what he wants. I know he wants to live”. (Family 4)  “Well, I’ve had a little more experience ‘cause I’ve been sick since 2008, so... that was with cancer to start with.... when you have cancer, the first thing you think of is: I’m going to die.... I’m not afraid to die. I’ve made that commitment to myself and decision that I’m not going to worry about dying. I’m going to focus on quality of life.”” You know some people... they hold onto people because they just can’t let go. And I wouldn’t want him to ever be like what happened to my step-dad [when he] was in that position. His wife held on to him for too long, and then finally let him go. But really, he laid there for two weeks and suffered, in my opinion. I can’t deal with that”. (Family 3)  “We were blindsided by the whole shock of being here, and then the shock of that conversation. That seemed to happen so quickly”; “Nobody likes to talk about death or dying, but it’s... less stressful talking about something before it happens, opposed to being on your death bed being asked to sign something.” (Patient 4)  “…and actually was really impressed with how calm and compassionate and open he was, which was really great.” |
| Ros et al 2021 | The positive and negative predictive values of the surprise questions for ICU admission, hospital admission, and 1-year survival were, respectively, 64%/94%, 59%/92%, and 60%/86%. Accordingly, the mean accuracy and kappa statistics were 93% (95% CI, 92–94%), κ equals to 0.43, 89% (95% CI, 88–90%), κ equals to 0.40, and 81% (95% CI, 80–82%), κ equals to 0.43 | **INFORMATION:** Clinicians are more accurate at predicting shorter-term survival (e.g. in ICU and hospital) and less accurate in in predicting 1 year survival.  **IDENTIFICATION**: physician has small but significant added value when incorporated with usual outcome prediction scores (OR 0.19) |  |
| Schonfeld et al 2012 | Themes  Compared to those with terminal illness more difficult to have EoL conversation because trajectory less clear and less able to rely on statistical evidence wrt prognosis. As a consequence, patients and families seem to believe that other diseases with comparable life expectancies (eg, heart failure or renal disease) can be treated or cured; more likely to seek 2^nd^ opinion. If QoL is poor, they tend to be more open to EoL discussions  Timing of conversations – facilitated by physical and/or social cues including: health status of the patient (especially significant weight loss), frequency of visits (outpatient or inpatient), efficacy of treatment, or decreased functional status. Age also plays a role in physicians willingness to initiate EoL discussion. However, often a lack of clear threshold or prompting event. Social cues: establishment of relationship/rapport with patient/family. Re timing – ideally before the patient gets sick and the earlier the better. They felt sickness only adds to anxiety felt by both patients and families confronting these issues. For this reason, some participants reported that, if there is potential for improvement in a patient’s condition, they will sometimes wait to get the patient back to baseline before initiating the conversation. They admitted that there is some confusion and uncertainty about the ‘‘right’’ time to initiate these discussions and that they often occur later because there is no obvious catalyst for the conversation. however, a physician may sense the need to broach the subject when a patient’s wishes seem to contradict medical reality (eg, the patient remains on full-code status despite declining over multiple hospitalizations). Also, participants mentioned that EOL issues may need to be brought up several times in order to prime patients and families to make decisions at a later date.  approaches to EoL conversation with MCM patients/families (cf those with terminal diagnosis). 3 approaches: i) Direct approach after clinician have gaugued “readiness”. However family may resist due to feeling of guilt or not accepting EoL is an option; ii) Indirect approach – physician attempts to get patient/family to bring up EoL conversation (e.g. by asking how they think the patient is doing); iii) Collaborative approach by formally calling a family meeting | **INFORMATION:** less available information on prognosis in those with MCM, which may increase likelihood of not accepting EoL situation or unrealistic expectations from patient/family  Conversations often immaterial if there is no patient acceptance or “buy-in” to the concept of EoL  **INFLUENCE:** Acute illness adds to anxiety faced all-round and from physicians point of view better to have EoL conversation either at “baseline” when stable or if there is a reasonable chance of recovery, better to wait until the patient has recovered.  **RELATIONSHIP:** prognostic uncertainty/unrealistic expectations on outcome – pts/families more likely to seek 2^nd^ opinion (with a potential detriment to trust in treating physician); Building a relationship/rapport with patient/family increases the likelihood of having EoL conversation and “priming” the patient family before so that they are ready to have meaningful conversations.  **SKILLS:** Clinicians find EoL conversation more difficult in patients who have MCM compared to those with a defined terminal illness.  patients/families (cf those with terminal diagnosis). 3 approaches: i) Direct approach after clinician have gaugued “readiness”. However family may resist due to feeling of guilt or not accepting EoL is an option  Indirect approach – physician attempts to get patient/family to bring up EoL conversation (e.g. by asking how they think the patient is doing);  iii) Collaborative approach by formally calling a family meeting  Acklowledging guilt and reassurance often helpful for surrogate decision maker  Empowering or giving permission patient/family to give input is often a facilitator to have these conversations  **IDENTIFICATION:** Lack of clear threshold or prompting event sometimes makes it challenging or confusing as to when to have the conversation. The consequence are that these conversations are often delayed. Physical cues: markers of deterioration health status (especially weight loss), frequency of visits to health services (as outpatient or inpatient); poor efficacy of treatment; decreased functional status; age; when patients wishes seem to contradict medical reality (e.g. patient remaining full code status despite frequent hospital presentations.  **ORGANISATIONAL:** more time often required in the MCM context to get patients/families to understand and accept EoL situation  Conversations don’t always happen because work environment is too busy/hectic  Organisational culture/expectation | “but the family sometimes and the patient feel like they need to pin it on something. It’s because of this specifically. And so it’s easier when you have . . . a specific diagnosis that you can say ‘This is the reason.”  “We can treat congestive heart failure. We can treat the decubitus ulcer. We can treat the renal failure. Each of these things in and of itself is potentially treatable if not curable, but when you put them all together and somebody who just seems to be falling like dominos and falling apart like a house of cards. We all know from our experience that person will not do well. But it’s really hard when you get a family member who says ‘You know, I’ve read up on this and a little antibiotics will take care of the pneumonia.”  “But people will seem to think there’s a cure for everything but cancer now. But people can somehow accept that ‘I have can- cer, I’m going to die.’ But everything else it seems like ‘Well, why don’t you fix that problem’ like ‘There’s medicines, there’s stuff to do.’ And they’re not nearly as accepting of any other diagnosis.”  ‘Do you want to know your prognosis?’ And if the patient says ‘‘no,’’ then that’s completely—that is a divergence point in your con- sultation. And there are some people who will flat out say ‘No, I don’t want to know. I want to know what I can do to beat this”  “[I]t’s just I guess super important to have these conversa- tions with patients before it gets to that point where it’s like ‘‘We need to make a decision now.’  “ Well, ideally it’s time before any of this comes up. We’re supposed to be doing this like the first time you see the patient, so that you don’t have to have an excuse to have the conversation.”  “It’s much easier to answer the family’s questions and the patient’s questions, too, when you have somebody who has stage IV lung cancer, you can say this is percent survival at this time and based on what your disease is rather than this conglomeration of all the things that are going wrong. It’s hard to predict for them how long they’ll be around and how to plan for things.” Also find it easier when patients have poor baseline function/quality of life. “And then you’ll have someone who—of the same age who has all these morbidities and has a terrible quality of life, so it’s kind of simpler talking to them because you can say ‘‘Look at Mom, look at Dad, they’ve got—this is their quality of life right now.’’ As opposed to someone else who is like ‘‘Well, I want to fight this’’ you know, even though they may have a terminal disease.”  “Do you want to know your prognosis?’ And if the patient says ‘‘no,’’ then that’s completely—that is a divergence point in your consultation. And there are some people who will flat out say ‘No, I don’t want to know. I want to know what I can do to beat this”  “I think some of the most successful conversations like this that I have seen have been preplanned, so whoever needs to get there has time to get there .... You kind of make it clear and we all agree on it as a group, so that we don’t leave the room and keep rehashing . … So we just make very clear decisions. And kind of make sure that everybody’s on the same page and give every- one a chance to bring things up if they have issues with what’s going on or being said and those kinds of things, and I think giving everyone a voice in the conversation.”  ‘It’s okay to do this and I know you’re going to feel guilty, but it’s okay to make this decision.’’ I think that it takes a big weight off people’s chests and allows them to even discuss it”  “And sometimes I think we just need to empower patients or families. It is okay to forego treatment, to forego burdensome treatment. Sometimes they don’t realize this.”  “Whereas, at what point do you have the conversation with your patient in the nursing home that has all these co-morbidities? And yes, this, at the time you establish the dementia diagnosis, you talk about a typical course for a dementia patient, although it’s extremely variable. But what, at what hospitalization, at what co-morbidity that pops up, do you then approach them and have this end-of-life conversation?”  ‘Well, this happened or when that happened, but that’s part of the process’ and to review this for them to help them create an image of that their parent or loved one is actually dying. So those are the pieces I see in the debility conversation that are critical and do take more time and more effort”.  “I grant you this often gets overlooked [EoL conversation] in the hectic pace of everything else we do.”  “Well, ideally it’s time before any of this comes up. We’re supposed to be doing this like the first time you see the patient, so that you don’t have to have an excuse to have the conversation.” |
| Shah et al 2016 | Facilitators of GoC discussion: use of simple language and exploration of previous experiences with LST  Barriers:  Patients symptoms (n=2)  Diagreement between family members and patient about code status (n=1)  Resident focuses on details of medical care. | **INFORMATION:** Using simple language; Previous experience experiences with LST were facilitators  Barriers were patients symptoms  Discordant outcomes from conversations – i.e. patient did not want to be intubated but doctor’s plan was “full code status” (resident interpreted the patient’s response of ‘do whatever you can’ to imply wanting all aggressive measures despite the patient declining intubation earlier in the conversation).  **INFLUENCE:** disagreement between family and patient about code status  **SKILLS:** In 12 out of 15 (80%) admission encounters, the resident initiated the discussion.  In 3 of 15 encounters, the topic of GoC was introduced but there was a missed opportunity to further engage the patient in the discussion.:  Resident focuses on details of medical care and not on life goals and the overall picture | “Have you given any thought as to whether you would want to go to the ICU, or have CPR, being on a breathing machine?” “I don’t want to be on a breathing machine. I just went through this with my mom, and I think this is why I’m having a hard decision. Because she passed away in April.”  “So every patient who is admitted, we ask the question, so don’t be afraid of anything. So just in case your heart stopped, or your breathing stopped, and we needed to do CPR and tube in, an intubation, and with ICU involved, would you like that? “; “Right now, yeah. So am I admitted?” “Right. Yeah, most likely, but I’m going to tell you the plan later. I’m going to examine you now.”  “So, the two options that I presented were number one, a more aggressive approach where we would connect you to a ventilator, where we would put the tube down your throat.” “No, I’m not for that.” ; “And, CPR would be entailed in that as well, and giving you those medications to artificially bring up your blood pressure. The other option would be just keeping you completely comfortable and giving you medications. If you were to start struggling, we would give you medications just to relax.”; “Well, it all depends what was wrong with me. Like today I don’t want anybody to think that I’m going to die. I don’t feel like I’m going to die.” “So, would it be safe to say that right now you would want us to be a little bit more aggressive?”; “To do whatever you can do, yeah.”; “So, we’ll keep you as a full code and we’ll do everything to keep you alive.” |
| Sharma et al 2014 | Themes identified included:  exploration of patient values/goals  framing code status as a patient decision, discussion of resuscitation outcomes and quality of life, and making a recommendation regarding code status.  Intervention residents were more likely than controls to explore patient values/goals (p=0.002) and make a recommendation (p<0.001); and less likely to frame the decision as one solely to be made by the patient (p = 0.01). Less than one-third of residents discussed resuscitation outcomes or quality of life.  Intervention had no difference with respect to describing resuscitation outcomes and discussing QoL | **INFORMATION:** Themes that appeared to influence the way in which residents determined code status. a) exploration of patient values and goals “What makes you happy, what makes your clock tick when you’re not acutely sick?” b) framing code status as solely a patient decision “I guess the decision’s absolutely and entirely yours.” c) describing resuscitation outcomes “Few people would survive a resuscitation attempt and be able to leave the hospital.” d) discussing quality of life “Some of these things will definitely prolong your life, but the quality of your life may not be as great as you may want.” e) making a recommendation regarding code status “My recommendation would be no, that you should not be what we call full code, you should be a do-not- resuscitate.”  Those where DNR was decided, tended to discuss more frequently about QoL following CPR  Compared to those who were full code, who were more procedure focussed and less on QoL following CPR  **SKILLS/TRAINING:** Residents in the training intervention arm explored patients values and goals more than compared to those in the control group (p<0.02), although they often didn’t integrate information given by patient into code-status discussions. In this example, patient was looking forward to returning to work as a writer, seeing his dog and getting some energy.  Training has the potential to modify the clinician-patient relation and lines of responsibility in decision making. Residents in the control group were more likely than those in the intervention group to frame the decision for code status as one that only the patient could make (57.1 % vs. 21.7 %, p = 0.01) even if the patient asked for advice or a recommendation  Compared to intervention when making a final recommendation was a more common occurrence (39.1% intervention vs 0% control  Training intervention had no difference with respect to describing resuscitation outcomes and discussing QoL. | “heart stop beating pounding on your chest type of event, and what I’m saying is in my experience those people are typically in an intensive care unit, rarely able to communicate with their family, certainly if they’re ventilated, it’s difficult to speak, I mean you can’t speak.”  “Sometimes that process can be pretty traumatic and it’s not always 100 %, you know, it doesn’t always work, but we would do all that in hopes of restarting your heart.”  “DOCTOR: Wow, okay, sounds good. Have you been doing any writing while you’ve been here? Patient: Not so much. DOCTOR: I’m sure it’s hard…. I just wanted to clarify a couple of things. One of them is in case of an emergency in the hospital have you talked to a doctor or even a family member about what you would want us to do in case of an emergency? Patient: No. DOCTOR: No, okay. So things like putting a tube down your throat to help you breathe, or antibiotics, things that would help prolong your life. Would you want us to do that while you were here?”  “No. It’s only a decision that you can make. And I could never tell you what to do because it’s a decision you have to make for yourself.”  “If it’s okay with you could I make a recommendation? . . . I think just from what I’ve heard from you in terms of your goals and everything, you would prefer to be as functional and active as you can be. And I’m just afraid that if you were to go through all of the things that we would have to do to try to resuscitate you that you wouldn’t get back to where you want to be. So, I think for, I mean, this might be a bit premature to say, but I think for you it might be more in line with what you want to go down the other pathway of saying, “please don’t resuscitate me.” But I’m not sure how does, when you hear that how does it feel?” |
| Sterie et al 2021 | Mean length of resuscitation conversations was 1 minute 45 seconds (SD = 96 seconds)  Conversations mainly focussed on technical procedures and rarely on outcome  With one exception, medical indication and prognosis of CPR did not feature in explanations  Explanations occurred either before the patients answer (as part of a question about CPR preferences or after patients answer which was generated by indecision, misunderstanding and the need to clarify answers. | **INFORMATION:** Mean length of resuscitation conversations was 1 minute 45 seconds (SD = 96 seconds)  Conversations mainly focussed on technical procedures and rarely on bigger picture.  Or no explanation of CPR  With one exception, medical indication and prognosis of CPR did not feature in explanations  Explanations occurred either before the patients answer (as part of a question about CPR preferences) or after patients answer which was generated by indecision, misunderstanding and the need to clarify answers. | Phy#15: (...) if the heart all of the sudden, it stops, the lung, Pat: Yes, it's my son [who decides]. Phy#15: Yes, but I mean, yourself, you wish us to do a resuscitation? That we try to restart the heart? Pat: I don't know, I don't know about this. I don't know at all. Phy#15: So, let's say that when we have a cardiac attack which... which... in which the heart stops. It's quite serious as complica- tion, so what we have to do is use perhaps some electric current to shock the heart, to put a tube to support the lung. Sometimes one recovers well, other times we can have side effects, or not re- cover or have side effects. This is something that is unpredictable. Pat: Yes, we can't know. Phy#15: We can't say what a person... we know that the longer we try to resuscitate... Pat: Yes, yes.Phy#15: The more possible it is that complications arise, but again we can't know... Pat: No, we can't know, of course. Phy#15: So, if it happened and if there was an attack, do we try to resuscitate? Pat: Yes try, yes try”.  “Phy#6: And just one question that we ask each patient who is hospitalized here, if your heart it stops, do you wish that we resuscitate you or not at all? Pat: Oh yes of course. Phy#6: All right.” |
| Strachan et al 2018 | Main themes:  The ambiguous nature of the nurses’ role in formal, physician-led, decision making and communication (as viewed by both physicians and nurses)  Most physicians valued nurses as part of discussion but competing work commitments meant that they weren’t always available.  Nurses play a key role in advocating patient wishes and preferences, especially in goals of care discussions and decision-making but usually on a more informal basis separate from discussion with the clinician.  Nurses said they were often called upon by patients or their families to clarify and make sense of information that was conveyed by a physician (CMO 1) | **INFORMATION:** Nurses said they were often called upon by patients or their families to clarify, simplifying and make sense of information that was conveyed by a physician  Facilitators during LST conversations  **RELATIONSHIP:** Nurses spend a lot more time with patients/families compared to doctors and the rapport and relationship they build eases some informal discussions taking place about LST  **SKILLS:** All nurses agreed that goals of care discussions and support of decision-making processes was within the nursing scope of practice, although they expressed variation in their ability and confidence to lead the team in these formal conversations.  Nurses advocate for patients  **IDENTIFICATION:** Nursing surveillance during patient care positioned them to notice and alert the healthcare team, essentially “raising the red flag” to the need for communication  **VALUE:** Most physicians valued nurses as part of discussion  **ORGANISATIONAL:** The ambiguous nature of the nurses’ role in formal, physician-led, decision making and communication.  Competing work commitments means that nurses aren’t always available.  Nurses were either invited by physicians into a conversation or a nurse happened to be there when such a conversation occurred with variable involvement.  Unspoken intentions on both nurses and physicians side which effects whether a nurse is present or not | “It's usually always been the physician that has that conversation and then (nurses) just to reinforce the conversation afterwards, because the family a lot of times is quite overwhelmed and then has a lot of follow-up questions afterwards. I think just reassuring them that just because they say they made the patient ‘do not resuscitate’ doesn't mean that they're not still going to be taking care of the patient or we aren't going to be still taking care of the patient or that they've given up.”  “They had had the discussion that he would not want to be on machines, okay? But then I explained that life sustaining measures go beyond just intubation, chest compressions, that there are other aspects. So when I explained this all to them that in fact giving fluids was life sustaining and that there is an option besides, any time something happens, to call an ambulance. They were actually extremely enthused about the concept of maintaining a palliative amount of care, right? So what they said was ‘Well we didn't really know that that was an option to us. We just want Dad to be comfortable.”  “It was great because I knew what all had been said and could follow through with her (the patient) and reinforce it and reassure her of what he (physician) had said” [nurse]  “We spend a lot more time. I might spend a week with a person, eight hours a day for four or five times in a week and so I think that amount of time and that kind of exposure and relationship sort of leads to more conversations sometimes.”  “My focus often in practice is with older people. I talk about more their wishes and sort of make it known more is this something you want to do? You know that you can always say no, because I find that lots of people don't really know that that's an option. They're offered a treatment but they're never really offered doing nothing versus something and I don't know if people are fully prepared for some of the suffering that they endure from the treatment itself and what the extent of the outcome of the treatment would be. So that's what I notice a lot that plans are offered but not a lot of plans or risks or benefits discussed about doing nothing versus something”  “They're not as responsive to us anymore, their appetite has decreased and they're not swallowing well. They're just sleeping more and more. And I often ask (the physician) ‘What's the plan for this patient?”  “I find that it helps with the nurse's presence there just because it's hard for me sometimes as the resident especially with the shift system with the night float to come on. I'm not on the ward so I haven't followed, I don't have a rapport with the family if they've been in hospital for quite some time. But the nurse is there day after day usually. So it's nice to have the nurse there just to be part of that conversation so they know the discussion that's had and can answer any of the patient's questions when I leave.”  “There are physicians who will grant that role to nurses, and others who will assume that role. And that's why we never know what our role is. [nurse]”  “I invite them in. Yeah, I invite them but often they're busy. They're just too busy. And sometimes the goals of care discussion can take an hour if there's a lot of... like we will often book family conferences to address it because it can't just be f!y-by, you know… I always invite the nurses to come but it's hard because they can't really take an hour o# their time. But sometimes they'll come. Usually I'll just let the nurse know on my way in ‘Hey, I'm going to talk to the patient about goals of care. Do you want to come?’ Or I'll say ‘I'm going to phone the family. I’ll let you know what they said”  “I will usually stand behind the curtain, and not go on the other side of the curtain and be present with the conversation that's happening (between a patient and physician). I'll just listen. I won't be a contributor in that conversation. I don't know why I do that”.  “I don't usually seek them out to bring them to those kind of discussions and I don't know if they would have time or interest. Like I've never had a nurse ask me that they want to come to a goals of care discussion”. [physician]; “(I am) not usually (involved) unless I kind of make it a point, unless I go ask a doctor and kind of happen to be there. But not necessarily. There isn't necessarily an intent or effort on the physicians to actually include us.” [nurse] |
| Sullivan et al 1996 | Timing of discussion re MV ideally when patient in a stable condition but usually introduced when acute deterioration +/- when too late  Importance of knowing the patient  Content of the discussion: often a well-rehearsed description of intubation and MV process including discomfort (9/15), inability to eat of speak (7/15), pain relief (13/15). Mentioning of death was not stressed by name initially but was the implied alternative if decision not to intubate  Framing the discussion:  15/15 final choice to be made by patient but 14/15 admitted framing the presenting information to influence patient choice. Framing usually around clinicians judgment. Initial description of intubation often explained in neutral way but then physician will often “lead” the patient  Decision difficulty: 7/15 thought discussions were difficult but necessary. Unpredictable trajectory. Difficult for patients to grasp and accept in short periods of time (14/15). Physician-patient discordant views more difficult; discussions easier when patients preferred physicians to decide of their behalf and easiest when concordant views. 12/15 family important to be involved  Style and delivery: Content similar but style and delivery of information varied between physicians. Either factual exchanges or personal, sensitive conversations | **INFORMATION:** content discussed included a tube being placed down a persons throat with emphasis on discomfort and not being able to eat or speak; reassured about pain relief regardless of choosing LST or not. Death not often stressed by name but was the implied alternative if patient chose not to be intubated  **INFLUENCE:** Framing usually around clinicians judgment. Initial description of intubation often explained in neutral way but then physician will often “lead” the patient.  **RELATIONSHIP:** “knowing the patient” was thought to be highly important.  Family involvement also thought to be important.  **SKILLS:** Discussions were viewed as being difficult, Worsened when patient-physician had discordant views. Eased with concordant views or when patients asked physicians to decide on their behalf). Content of information provided was often similar but the way information was delivered varied  **IDENTIFICATION:** Timing of discussion re MV ideally when patient in a stable condition but usually introduced when acute deterioration +/- when too late. Unpredictable trajectory made things more difficult to judge when was the right time to have the conversation  **VALUE:** Conversations were regarded as being necessary | “but we don't stress too much the word "death" or "dying." I think it becomes evident as we speak”  “You might color it one way ot the other, wealways do. There are some people who you just know are bad intubation candidates and for those people I'll paint a bleaker picture.”  “I never bring it up with people I've seen only once.” |
| Syed et al 2017 | Family-related barriers were found  to be the most common barriers. They include family denial (74.0%), level of education of family (66.2%), and conflict between individual family members (66.2%). Regarding personal barriers, lack of knowledge regarding prognosis (44.1%), personal discomfort in discussing death (29.8%), and fear of legal consequences (28.5%) were the top most barriers. In hospital-related barriers, time constraint (57.1%), lack of hospital administration support (48.0%), and suboptimal nursing care after DNR (48.0%) were the most frequent. There were significant differences among opinions of trainees when compared to those of attending physicians. | **INFLUENCE:** Family related barriers: Family denial (74%), level of education of family (66%), family thoughts that physician wants to take off their hands from the patient 54% (trainees) and 28% (attending) and patient or family member are doctors (55%) language barrier of family (52%); conflict between individual family members on decision of DNR (66%), secondary gains by family (31%)  **TRUST/RELATIONSHIP:** Fear that raising topic of death would compromise doctor-patient relationship (16.9%); families do not take recommendation by physician (29%)  **SKILLS/CONFIDENCE:** Lack of knowledge regarding prognosis (44%), personal discomfort discussing death (30%) and fear of legal consequences (29%) (more for trainees compared with attendings), fear of family reaction (27%); Lack of training in communication skills (10.4%); Doctors attitude that they are a symbol of hope not death (20%) or taking away their hope (14%), code status should be discussed with patient not family (42%)  **ORGANISATIONAL:** There were differing views as to who is responsible to have these discussions (i.e. primary responsible physician or me) 22%; Organisational barriers for code status discussions: time constraints (57%), lack of hospital administration support in case of family reaction (48%), poor nursing care after declaring DNR (48%), poor care by doctors after declaring DNR (38%). Trainees considered all 3 barriers more as a barrier compared to attending physician; Lack of proper place for code status discussion (46%), Hospital policies (35.1%); Lack of trained nurses (32%) |  |
| Taylor LJ et al 2018 | Despite decision aid, some themes still remained:  Surgeons frequently missed an opportunity to break bad news. By focusing on the acute surgical problem and need to make treatment decision, surgeons failed to expose the life-limiting nature of the patient’s illness  Surgeons asked patients to express preference for a specific treatment without gaining knowledge about the patient’s priorities and exploring how patients might value specific health states or disabilities  Many surgeons struggled to integrate patients goals and values to make a treatment recommendation. Instead they presented options and noted “it’s your decision” | **INFORMATION:** Focus on acute surgical problem and not the overall picture nor prognostication E.g.92 year old, frail, colon cancer  Surgeons asked patients to express preference for a specific treatment without gaining knowledge about the patient’s priorities and exploring how patients might value specific health states or disabilities  Often focussed on survival vs death with little discussion about anything in-between (i.e. QoL in relation to patient values).  **SKILLS:** Many surgeons struggled to integrate patients goals and values to make a treatment recommendation. Instead they presented options and noted “it’s your decision” | ‘‘It’s possible that despite our best efforts that we couldn’t get over it and that we couldn’t get you healthy after the operation.” [but no mention of overall picture/bad news/EoL]; ‘‘That’s why we’re recommending just amputating the foot. It will remove the infection and give him a chance to come back from the infection.’ [90 year old patient with gangrene]  ‘If this blockage keeps up like this, do you want to just let it go and die from it or would you rather have an operation to try to fix it?’’; MD: ‘‘And have you thought at all about what you would want?’’ Patient: ‘‘Well I’d like to survive’’ MD: ‘‘Well that’s good cause then we both want the same thing. That’s great...Well I think we should go forward with the surgery.’’  Patient: ‘‘I don’t know what to do, doctor’’ MD: ‘‘No, it’s up to you. . ..you could just think about it.’’ |
| Thomas et al 2021 | SDM goes beyond mutual understanding between patient and clinician. The patient understanding the benefits, burdens of treatment options and prognosis and the clinician understanding patient values and their opinion.  Other things to consider:  The effect of heuristic mode of decision making vs rational particularly in situations that are time sensitive. This reduces the liklihood of SDM  Risk perception on the part of both patient and clinician; positive and negative framing  Influence of race, ethnicity and cultural backgrounds may influence the degree of trust and efforts to make personal connection  Instability of preferences and the impact of emotions can adversely effect the patients to fully consider benefit-risk trade-offs. This may lead to decisions based on feelings v information  Clinician level: unnecessary jargon, providing too much information, presenting information in a biased manner; clinicians emotional experiences (e.g. fear may cause clinicians to withhold information due to concerns about causing harm); overemphasis of options/tyranny of choice can result in psychological harm rather the effective decision making  Organisational level: time contraints 🡪 heuristic decision making (see above); lack of continuity of care  Recommendations  Patient level: consider impact of culture, expectations and information needs; deduce who are decision influencers for this person and how should they be included in SDM process; how are experiences and circumstances likely to affect decision making  Clinician level: awareness of own influences in SDM process; relational v transactional model of consultation process  Systems level: SDM not a tick box exercise – assessment of individuals decision making needs and preference as well demonstration of effective communication and relational practices should be incentivised; follow-up to ensure better chance of stronger clinician-patient relationships | **INFORMATION:** Clinician level: unnecessary jargon, providing too much information, presenting information in a biased manner (e.g. fear may cause clinicians to withhold information due to concerns about causing harm); overemphasis of options/tyranny of choice can result in psychological harm rather the effective decision making  **INFLUENCE:** The effect of heuristic mode of decision making vs rational particularly in situations that are time sensitive. This reduces the liklihood of SDM; Risk perception on the part of both patient and clinician; positive and negative framing; Instability of preferences and the impact of emotions can adversely effect the patients to fully consider benefit-risk trade-offs. This may lead to decisions based on feelings v information  **TRUST/RELATIONSHIP:** Influence of race, ethnicity and cultural backgrounds may influence the degree of trust and efforts to make personal connection  **SKILLS/CONFIDENCE/TRAINING:** Patient level: consider impact of culture, expectations and information needs; deduce who are decision influencers for this person and how should they be included in SDM process; how are experiences and circumstances likely to affect decision making; Clinician level: awareness of own influences in SDM process; relational v transactional model of consultation process with focus of relational communication skills training  **ORGANISATIONAL:** Organisational level: time contraints 🡪 heuristic decision making (see above); lack of continuity of care; Systems level: SDM not a tick box exercise – assessment of individuals decision making needs and preference as well demonstration of effective communication and relational practices should be incentivised; availability of time and follow-up support to ensure better chance of stronger clinician-patient relationships |  |
| Tulsky et al 2017 | 5-areas identified in which further research could move the field forward:  Measurement and methodology (including how to determine communication quality)  Mechanisms of communication such as identifying the specific clinician behaviours that patients experience as both honest and compassionate, or the role of bias in the clinical encounter  Alternative approaches to advance care planning that focus on the quality of serious illness communication and not simply completion of forms  Teaching and disseminating communication skills  Approaches, such as economic incentives and other clinician motivators, to change communication behaviour. | **INFORMATION:** Patients generally want prognostic information Prognostic information is often neglected or given in optimistic terms as clinicians worry about causing distress and loss of hope, which is contrary to evidence that this does not happen; Shared-decision making is not defined and can be variable and studies have shown that physicians frequently fail to discuss options robustly or elicit patients’ values in decision-making encounters. More research is needed to define shared decision making, observe how it unfolds within clinical encounters, and determine how patient attributes affect the shared decision-making process.  **INFLUENCE:** Patients with cognitive impairment often excluded from trials and therefore their needs with regards to communication have been under-researched. Emotions are likely to influence the processing and understanding of information that is being conveyed. It is important to learn how much and what kinds of emotions affect decision making, and how the effect of emotions on decision making varies among patients. How do patient emotions change over time, and how does the quality of the communication influence these changes? And how can physicians facilitate patient emotional expression and involvement? The influence of culture is also underresearched - it is not clear how cultural competency differs from patient-centered communication. It is not known how clinicians can best communicate with patients with various cultural and individual preferences. Nor is it known which communication strategies work best to assess patients’ needs and tailor care to the individual patient within their cultural context; the effects of social media and other external sources in how they may bias patient/family perceptions needs exploring further  **SKILLS:** Multiple approaches for training in serious illness communication skills including face-to-face workshops, feedback on actual medical encounters both live and audio-recorded, online interactive courses, and innovative computerised simulation approaches. These can positively impact clinician behaviour but little is known about the long-term effects of this training, the intensity of interventions that are necessary for success, the value of follow-up skills training, how to engage unmotivated learners, and how to bring such programs to scale. Future directions to be explored: ongoing training and maintenance of certification of communication skills, develop quality measures for communication and rewarding clinicians who achieve specified communication goals, such as documentation of the conversation, minimal scores on patient surveys, or even quality as determined by audiorecorded encounter.  Measuring quality of communication: dimensions include verbal and non-verbal words, actions and behaviours during a clinical encounter and how those words/actions/behaviours are understood and perceived. Outcomes include patient trust, satisfaction, decision quality and health care use  **IDENTIFICATION:** The value of EHR prompts need to be explored further  **VALUE:** More research needed into clinician motivation strategies (including incentivising) to engage more with patients and families |  |
| Uy J. et al 2013 | 38/98(39%) physicians sent the patient to the ICU, and 9/98(9%) ultimately decided to intubate. Most (93/98 (95%)) provided at least some medical information, but few explained the short-term prognosis with (26/98 (27%)) or without intubation (37/98 (38%)). Many (80/98 (82%)) elicited the patient's intubation preferences, but few (35/98 (36%)) explored the patient's broader values. Physician-patient relation described: 1/98 (1%) as informative, 48/98 (49%) as facilitative (SDM), 36/98 (37%) as collaborative, and 12/98 (12%) as directive (paternalistic); 1/98 (1%) could not be placed into a category. No observed physician characteristics predicted decision making role. | **INFORMATION:** 95% explained the medical condition; 37% explained more than 1 treatment option; 27% mentioned possible outcomes with intubation; 38% mentioned death likely if intubation withheld; 69% prognostic statements not tied with any treatment plan; 43% attempted to ascertain what patient/surrogate knew about cancer or to discuss long term prognosis if they survived critical illness  **INFLUENCE:** 10% physicians guided the surrogate to separate their own personal wishes from what the patient would choose for themselves, with priority given to the patient’s own wishes.  13% highlighted the preference-sensitive nature of decision to initiate LST For example, in response to a surrogate’s request for a treatment recommendation  **RELATIONSHIP** (type):1% informative: provided information (medical condition/prognosis/treatment options) but did not seek information or provide any recommendations about care; 53% facilitative – no recommendations but actively guided patient and surrogate to clarify patient’s values and apply those values to decision-making; 33% collaborative - actively participated in deliberations with the family, elicited patient values and provided a recommendation based on these values. 12% physicians directive - made an independent treatment decision without considering the family’s values. One (1%) could not be categorised (brief history and then left the room without a plan).  **SKILLS:** 82% aimed to ascertain treatment preferences without relating to broader life term goals and patient values  Few physicians (4%) discussed the likely outcome of different treatment strategies prior to beginning a discussion about patient preferences.  78% encounters, the physician made an explicit treatment recommendation:  65% physician recommendation after solicitation from surrogate – half physicians responded to this request; 25% reframed the question back to the patient/surrogate; 31% restated the medical information; 14% ultimately refused to give any recommendation; 13% made treatment recommendations without solicitation from surrogate  9% stated their plans to intubate without seeking views of patient/surrogate | “Once more you are speaking for him right now, your decisions. What would he want if he was able to speak for himself in this situation?”  “That is a very difficult question. It is a very personal thing.”  “If we are not able to get you better with this mask or with some other noninvasive types of masks that provide oxygen do you want us to place a breathing tube to help you breathe?” |
| Vaderhaeghen, Van Beek et al 2019 | Systematic synthesis of qualitative studies. 23 studies included | The topic of focus during such discussions can be influenced by baseline speciality (e.g. internist discussing more about life goals compared to intensivist discussing more about medical intervention/code status); also variation in what ACP is. Some see it with filling a form of making a specific decision as the an end-point, others view it as a continuous process  Physicians included in different studies, stress the importance of being able to build up a relationship with patients and families. Taking time an important mediating factor  Physicians had difficulties with dealing with emotional responses of families and patients during conversations. Reasons were multiple. They comprised having not enough communication skills and having too much involvement emotionally when families or patients were going through a hard time. Many believed that, if they empathized too much with the family, their own personal emotional burden would be too great  The impact of emotion relating to: overcoming the fear of dying, fear of hurting patients, fear of causing undue suffering to families and dealing with stress and anger of patients’ families. Physicians reported that fear of hurting was a reason why they shied away from confronting patients with their unrealistic expectations  Medicolegal issues: Physicians report being hesitant about withholding life-prolonging interventions and chose invasive treatment because they fear legal repercussions  Lack of mental capacity often a barrier with difficulty in trying to work out what the patient might have wanted  Focus of conversation: too much on medical aspects/too procedure focussed (often with jargon/failure of patient/relative in understand) and less focus on personal values  Cultural barriers  Written agreements: seen as both helpful and as a barrier. Helpful as ease of references and to aid inter-professional communication but barrier because may not be specific to the situation which may be complex and may be subject to misinterpretation of decisions by family  Lack of experience is a barrier but also the important to learn from experience and improvement of skills happens over time  Framing: when families have a certain perception about decision- making, physicians report that they reframe those difficult decisions, to help families make difficult choices.  Clinicians thoughts about what type of physician-patient relation is most appropriate. Those who believe in SDM approach may be reluctant to give their opinion/clinical opinion  Having ACP conversations gives discomfort for clinicians particularly as it may lead to uncomfortable conflict or being blamed by family for not saving the patient  Not always clear who is responsible for having ACP conversations and whether this was their role. Also active v passive clinicians | ‘I think it’s hard to be a good doctor when you are so emotionally involved or personally connected to folks, because it clouds your decision-making and you will burn out before you even finish your training, being emotionally burdened by the decision making process’  I’ve sort of been taught it throughout medical school that ... we’re evidence based and we’re not paternalistic anymore, and you don’t tell people what to do .... People have different religions, different backgrounds .... I feel like it would be wrong of me to impose my views on them because ... everybody is so different  ‘You don’t even know how it happens, but it does. I guess that’s why they push you into the ward and you have to deal with everything yourself as an intern, because that’s the way you learn’  ‘If they ask for our opinion, I don’t think there is anything wrong in giving our opinion. But I think ... you should be a little cautious when you do that, just because you don’t want to influence anybody’; But also some clinicians with introduce bias by framing conversations in a certain way or omitting information with aim of getting the patient/relative to agree with their clinical recommendations |
| Vanderhaeghen et al 2019 | 4 “fields of tension”   1. Not knowing what to expect from the treating physician 2. Not being sure that the treating physician can be trusted as a partner 3. Daring to speak about ACP   Staying loyal to one’s own wishes | **INFORMATION:** Focus of conversation: too much on medical aspects/too procedure focussed (often with jargon/failure of patient/relative in understand) and less focus on personal values  **INFLUENCE:** Physicians had difficulties with dealing with emotional responses of families and patients during conversations; The impact of emotion relating to: overcoming the fear of dying, fear of hurting patients, fear of causing undue suffering to families and dealing with stress and anger of patients’ families. Physicians reported that fear of hurting was a reason why they shied away from confronting patients with their unrealistic expectations; Medicolegal issues: Physicians report being hesitant about withholding life-prolonging interventions and chose invasive treatment because they fear legal repercussions; Lack of mental capacity often a barrier with difficulty in trying to work out what the patient might have wanted; Cultural barriers:  Framing: when families have a certain perception about decision- making, physicians report that they reframe those difficult decisions, to help families make difficult choices. some clinicians with introduce bias by framing conversations in a certain way or omitting information with aim of getting the patient/relative to agree with their clinical recommendations  **TRUST/RELATIONSHIP:** Physicians included in different studies, stress the importance of being able to build up a relationship with patients and families. Taking time an important mediating factor. However, Many believed that, if they empathized too much with the family, their own personal emotional burden would be too great  **SKILLS/CONFIDENCE:** Lack of experience is a barrier but also the important to learn from experience and improvement of skills happens over time  Having ACP conversations gives discomfort for clinicians particularly as it may lead to uncomfortable conflict or being blamed by family for not saving the patient  **VALUE:** The topic of focus during such discussions can be influenced by baseline speciality (e.g. internist discussing more about life goals compared to intensivist discussing more about medical intervention/code status); also variation in what ACP is. Some see it with filling a form of making a specific decision as the an end-point, others view it as a continuous process; Not always clear who is responsible for having ACP conversations and whether this was their role. Also active v passive clinicians  **ORGANISATION:** Written agreements: seen as both helpful and as a barrier. Helpful as ease of references and to aid inter-professional communication but barrier because may not be specific to the situation which may be complex and may be subject to misinterpretation of decisions by family | I’ve sort of been taught it throughout medical school that ... we’re evidence based and we’re not paternalistic anymore, and you don’t tell people what to do .... People have different religions, different backgrounds .... I feel like it would be wrong of me to impose my views on them because ... everybody is so different  ‘I think it’s hard to be a good doctor when you are so emotionally involved or personally connected to folks, because it clouds your decision-making and you will burn out before you even finish your training, being emotionally burdened by the decision making process’  ‘You don’t even know how it happens,but it does. I guess that’s why they push you into the ward and you have to deal with everything yourself as an intern, because that’s the way you learn’ ; Clinicians thoughts about what type of physician-patient relation is most appropriate. Those who believe in SDM approach may be reluctant to give their opinion/clinical opinion ‘If they ask for our opinion, I don’t think there is anything wrong in giving our opinion. But I think ... you should be a little cautious when you do that, just because you don’t want to influence anybody’ |
| Visser et al 2014 | Barriers relating:  to lack of physicians knowledge and skills relating to lack of training in management of patients/families of EoL (including communication with them)  Physicians attitudes  Physicians practice  Stronger evidence was found for physicians’ lack of communication training and skills, their attitudes towards death in the ICU, their focus on clinical parameters and their lack of confidence in their own judgment of their patient’s true condition | **INFORMATION:** Clinicians feel that prognostic uncertainty is difficult to manage and communicate  **INFLUENCE:** The culture/views of different specialties may influence how clinician responsible communicates with patient/relatives (e.g. surgeon focussing on survival after operation and not from patient perspective); Views of the doctor than death is a medical failure;  **TRUST/RELATIONSHIP:** Relatives who do not understand or who have unrealistic expectations results in negative attitudes towards them by clinician which can result in avoidance of communication; decisions to forgo LST can lead to the feeling of abandonment by patient/relative (and sometimes anger/loss of trust), particularly if the focus of alternative treatment is not discussed.  **SKILLS/CONFIDENCE:** Physicians feel under-trained in dealing with communication with patient/relatives about EoL issues. Leads to feeling of not being comfortable or at ease with having such discussions  **IDENTIFICATION:** Too much focus on physiological and sometimes lack of recognition of dying process  **VALUE:** clinicians preferring a paternalistic model of care reduces likelihood of communication; Clinicians may feel that communication is a “waste of time” if family have certain views/unrealistic expectations that can be v time consuming; clincians also find it easier to carry on with LST rather than discuss withholding/withdrawing LST related to concerns about omission of life-sustaining treatment are larger (missing something treatable, fear of doing something wrong or limiting life-sustaining treatment for a patient who might survive) than concerns about harm of administering life-sustaining treatment (such as iatrogenic harms, prolonging dying, and treating patients against their preferences)  **ORGANISATION:** differences in opinion as to who is overall in control/responsible for patient in shared care situations (e.g. ICU/surgeon) can influence what is discussed with patient and family; Fear of legal ramifications if clinician do not do what the family want. |  |
| Vitale et al 2020 | Effect of fake news  False hope, Loss of trust in care team, Loss of attention to physicians words, Tension towards care team, Contrast medical decision  Potential countermeasures:  Gently deconstruct alternative truths providing credible “official” data and information. Always report the same information.  Do not create “fights” and do not put in contrast the “alternative truths” with physicians’ one. Instead, support the scientific views with data and reliable sources. Avoid letting relatives think of themselves as a “victim”.  Create a “scenography” around the physician with medical books, information sheets and official sources.  Let relatives understand that the care team is needed to provide best care and less suffering to their beloved.  Gently explain that it is not the idea of a single physician but of the whole care team, which consist of humans that try to provide best care according to current science and ethics. | **INFORMATION:** To counteract false hope, gently deconstruct alternative truths providing credible “official” data and information. Always report the same information  **INFLUENCE:** Fake news can be responsible for: false hopes, loss of attention to physicians words and disagreements with physicians opinion. Furthermore, Fake news may also be the trigger or exacerbate a situation where relatives’ desire to snatch clinical news from different medical consultations, hoping to get a custom-made truth, which better fits emotional desires regardless of their positive or negative value  **TRUST/RELATIONSHIP:** Fake news has the potential to undermine patient/family trust in medical team and has the potential for increased unwarranted tension towards the care team |  |
| Weigl et al 2009 | Hospital physicians spent 25.5% of their time at work in direct contact with patients. Most time was allocated to documentation and conversation with colleagues and nursing staff. Physicians performed parallel simultaneous activities for 17–20% of their work time. Communication with patients, documentation, and conversation with colleagues and nursing staff were the most frequently observed simultaneous activities. Applying logit-linear analyses, specific primary activities increase the probability of particular simultaneous activities. | **ORGANISATIONAL:** Surgeons and internal medics only spend 25.5% of time with direct patient contact of which 8% (surgeons) and 9.8% (internists) of total available time is spent directly communicating with patients/families. Lots of competing pressures of in-direct patient contact work including documentation (27 and 33% surgeons/internists respectively – which represented the highest proportion of non-patient facing work undertaken), communication with staff and others, organising and attending meetings, CPD and personal activities |  |
| Wubben et al 2021 | Main struggles with ICU physicians include uncertainty regarding long term health outcomes, time constraints, feeling pressure because of having final responsibility and fear of losing control  Former patients and family members stated aspects that they missed such as not feeling included in ICU treatment decisions and a lack of information about long-term outcomes and recovery  ICU nurses reported mainly opportunities to strengthen their role in incorporating non- medical information in the ICU decision-making process and as liaison between physicians and patients and family | **INFORMATION:** physicians commonly reported struggles relating to uncertainty of long term health  varying degree of prioritisation of medical facets in discussions by physicians with often less attention to “softer” topics such as QoL  Physicians often reverted to what they viewed was a good QoL without finding out whether similar values were shared with the patient.  **INFLUENCE:** Clinicians felt that if families too involved in ICU decision making, then there would be more “pointless” treatments.  **RELATIONSHIP:** Treating patient like a human rather than a “mystery illness fosters a positive relationship. “PF: Treating me as a human being instead of a patient with some mystery illness—yes, I would have appreciated that very much, especially now looking back.”  Patients open to being included in decisions. They cited that feeling heard and included was the most important factor, rather than having an equal hand in every and all decisions  They often did not want to have the final responsibility in life-death decision making, particularly with older patients.  **SKILLS:** clinicians often feared losing control of situations where they actively engage with patients and families about decision making and patients/families have “irrational wishes  **VALUE:** Difference between starting and withdrawing once started. Physicians feel that can always stop treatment further down the line which can limit conversations  **ORGANISATIONAL**: Nurses seen as facilitators to SDM process. Often seen as a liaison between ICU physician and patient/family. Help patients understand some of the jargon used by physicians and in turn are advocates for the patient by helping to promote patients values and broader life priorities to physicians and give context to physician | outcomes Physician (P): Overall I think there is an understandable tendency to postpone [decision making about] problems.(...)Again, the problem is always that uncertainty. The other day we lost a patient after 6 weeks of treatment.(...)On the one hand you can say that we all saw that coming. On the other hand—well, you only know for certain if you’ve tried it.  P: Look, in the end we all prefer talking about the fluid balance and CRP levels. That’s the truth. So [talking about quality of life] is ‘soft’ drivel to many people.  “P: What I used to see, and still see a bit—is that we physicians have our own opinions about what constitutes a good quality of life—in other words, what a good outcome looks like. And we do not look at the patient well enough”  “But if you wanted to optimize it, in my experience, you can tell people: what are you doing, why are you doing it. Even if people are half-conscious, you don’t know what they will remember. I think they are very much in a ‘state of doing’”  P: (...)especially older people say: no, you’ve studied for this—I don’t know, you tell me. They just put everything back in your hands. You want to have those people decide for themselves but they’re not going to.  ”…That you’re scared of promising something you can’t fulfill. It’s weird to then not ask the question, but that is a way of doing things. Or fearing totally irrational wishes from people.”  “P: At the same time it’s easier for me to intubate,(...)to start renal replacement therapy—far easier than not starting treatment. So I think that’s an important point.(...) Sometimes we use the multidisciplinary discussion to say to each other: are we really still on the right track?(...)And then you sometimes get one-liners like: ‘You can always stop [treatment), the patient can always say that they don’t want it like this [at a later stage).”  “ICU Nurse (N): Then after the family conference, you let it sink in, and you start repeating it and repeating it. And you try to use the same words as the physician—because I’ve noticed families say: I think it’s so difficult, one says this and the others says this – but that’s because [families] don’t understand. N: That is kind of the role we take on: [translating the family’s wishes for the physicians. N: Yes—[nurses] think it’s important to be of value in decision moments. Continuing or not, you know. Of course you need to do so based on medical information, but also based on the holistic view, and I think we should play a larger part in that, because we also know the family really well.”  Time barrier: “P: The limits are mostly put on by time and space. Sometimes you have a really busy day so you don’t have time for it. Then you need to cut back a little on those conversations, because there isn’t any time.” |
| You et al 2019 | Acceptbaility: 65/71 participants gave the CPR video a good to excellent overall rating and indicated that the tool provided the right amount of information in a balanced way, was clear and helpful and was a tool that they would highly recommend to others  Effectivenss: The intervention was associated with an improvement in knowledge about CPR (+2.7 points, 95% confidence interval [CI] 2.2 to 3.3, effect size 1.5)  29 patients and 20 family members (49/71) had complete responses before and after intervention (therefore 31 had incomplete data and were not analysed). Total mean score reduced from 25.7 to 7.7 (change -18.1;95% CI -21.8 to -14.3) with effect size 1.3. Largely attributed to informed and values clarity subscores (no significant effect on uncertainty subscore).  36/71 (51%) had a discussion with a physician about CPR after watching the video and rated the extent of shared decision-making as 6.3 (SD 1.7) (possible maximum score 9).  There was a nonsignificant decrease in the proportion of patients with a medical order for CPR after the intervention (71% before v. 63% after, p = 0.06). | **INFORMATION:** Dissemination of information relating to CPR (the technical aspects of the procedure itself and post resuscitation care on the ICU, short term and long-term outcomes was found acceptable to patients, improved their knowledge and aided their decision making capabilities. Regarding acceptability, the majority felt it provided the right amount of information in a balanced way that was clear and helpful. |  |
