# Supplementary material for: Examining how goals of care communication are conducted between doctors and patients with severe acute illness in hospital settings: A realist systematic review
Source: PLoS One. 2024 Mar 18;19(3):e0299933. doi: 10.1371/journal.pone.0299933 (PMC10947705; doi:10.1371/journal.pone.0299933)
Supplement: S4 Appendix — (PDF) [file pone.0299933.s006.pdf]

A realist review examining how wishes of patients with severe acute illness are formulated and communicated concerning life-sustaining care in acute hospital settings

Citation

Jamie Gross, Jonathan Koffman. A realist review examining how wishes of patients with severe acute illness are formulated and communicated concerning life-sustaining care in acute hospital settings. PROSPERO 2021 CRD42021297410 Available from: https://www.crd.york.ac.uk/prospero/display_record.php?ID=CRD42021297410

Review question

Our preliminary review question is: "What factors influence how patients formulate and communicate their wishes in relation to life-sustaining treatments in the context of acute illness in the hospital setting?"

Searches

Literature searching in realist reviews is an iterative process. As such, our initial systematic search will be revised throughout the review process and subsequent literature searches will be guided by the data. Systematic searches for published literature will be performed using the following databases: MEDLINE, CINAHL, British Nursing Index PsycINFO, Scopus, Web of Science and the Cochrane Library. In addition, papers will also be identified by citation tracking from papers already identified from the initial search and other search engines (e.g. Google Scholar). Hand searching of relevant journals will also be performed and we will contact disciplinary experts (in intensive care, palliative care, care of the elderly, medical ethics and resuscitation) to identify salient published and unpublished manuscripts and relevant websites. Grey literature will be sought from the openSIGLE website. We will not restrict the search by publication period. Only English articles will be selected. Those published in any other language will be excluded.

Types of study to be included

We will include all qualitative or quantitative study designs and other forms of academic and grey literature including literature reviews, editorials and guidelines

Condition or domain being studied

Patients that are admitted to hospital with acute illness face an uncertain outcome. Those who develop severe life-threatening illness in acute illness may be admitted to Intensive Care Unit (ICU). Receiving invasive organ support on the ICU can be unpleasant and prolonged ICU admission is associated with a higher long-term mortality risk, long-lasting physical weakness and psychosocial morbidity (e.g. Post Traumatic Stress Disorder) that can result in a lower quality-of-life. Healthcare professionals who play a role in making escalation decisions in acute illness should critically consider the likelihood to benefit from organ support against the potential burdens that are associated with receiving such care, whilst also being in accordance with a patient’s wishes and values.

Evidence shows that patients are rarely consulted in such situations. Moreover in circumstances where this does take place, little is known about how they arrive at and then express their wishes. This realist review will aim to explore these factors and what contribute to them in order to enhance understanding and ultimately look for ways in how to promote patient’s wishes being represented in the decision-making relating to life-sustaining care.

Participants/population

We will include articles relating to people who are hospitalised for acute illness and are at risk of or who show signs of

worsening acute illness. Articles will be selected based on their relevance to decision making relating to proceeding or not proceeding with life-sustaining care and patient involvement in this process. Focus will be largely centred around patient wishes relating to decisions made in relation to life-sustaining care in the acute hospital setting but where relevant, the community setting will also be considered.

We will exclude articles that focus on:

- patients under 18 years of age

- the terminal phases of life-limiting conditions

- other areas that do not involve decisions relating to proceeding or not proceeding with life-sustaining care

- Articles not published in English

According to realist review methodology, subsequent searches will be guided by the data; therefore we will revisit and potentially revise these inclusion and exclusion criteria and the review progresses.

Intervention(s), exposure(s)

Source articles will be included if they refer directly to decision-making relating to life-sustaining care (including DNACPR) or palliative care in patients that are either highly likely to face or are faced with severe acute illness.

Articles must include reference to patients wishes and will be excluded there is no reference to these.

Comparator(s)/control

Not applicable

Context

We will include articles that are based in the acute hospital setting but where relevant, the community setting will also be considered.

Main outcome(s)

Our main outcome is to to identify and examine factors that influence how patients derive their wishes relating to undergoing life-sustaining care interventions in acute illness and how these wishes are subsequently communicated and made accessible to clinicians that are involved in making such decisions.

Additional outcome(s)

No applicable

Data extraction (selection and coding)

Two reviewers will independently screen and select articles for inclusion. Articles will be considered for inclusion based on the question: "Does this data source provide any evidence, discussion or conceptual/theoretical perspectives that will enable us to test and refine our understanding of how patients formulate wishes relating to life-sustaining care and/or factors that influence how such wishes are communicated to clinicians involved in decision making for life sustaining care?"

Two reviewers will independently extract data using a tailored form that includes title, first author, year, country, study design and how, precisely the article contributes to the review question, CMO configurations and/or middle range theory. Two researchers will independently code data both inductively, to capture potentially relevant data based on the

researchers interpretations, and deductively based on a coding frame developed after identification of the provisional middle range theory and context-mechanism-outcomes (CMO) configurations. The coding frame will be revisited and revised as data extraction progresses.

Data interpretation will be guided by the reviewers' judgement; any discrepancies will be resolved by discussion until consensus is reached. If consensus us not possible, the reviews will consult relevant stakeholders/experts in this field.

Risk of bias (quality) assessment

This review supports Pawson theory of rejecting the 'hierarchy of evidence' approach to quality appraisal as this is incompatible with realist philosophy[1]. Instead articles will be assessed according to relevance (does the article address the theory under investigation?) and rigour (is the evidence reported in the selected article reliable and trustworthy?) The data extraction form will include an appraisal assessment section, on which reviewers will pragmatically appraise articles based on their usefulness and relevance to the review, rating their relevance as high, moderate, low or none. In addition, both researchers will keep a data diary of decisions made during the data extraction and appraisal process.

Studies might not be excluded due to poor quality. Flawed studies with relevant findings will be discussed on a case-by-case basis by the research team to consider their inclusion in the review

[1] Pawson, R., et al. (2005). Realist review-a new method of systematic review designed for complex policy interventions. Journal of Health Services Research Policy, 10(21), 21-34.

Strategy for data synthesis

Data synthesis will centre on testing and refining the theories with the ultimate aim of creating a theory that explains i) how patients formulate their wishes relating to life-sustaining care (including but not exclusive of their understanding of the possible treatments faced, likely outcomes, trade-offs and uncertainties) and ii) factors in healthcare that influence how patient wishes are communicated to healthcare professionals who are involved in making decisions relating to life sustaining care.

Using context-mechanism-outcome configurations, the researchers will explore the available evidence that helps support theory building - with focus on improving understanding of the factors and processes that explain how patients wishes are formulated and expressed within usual clinical practice. Evidence for factors that facilitate or inhibit patient wishes being communicated to relevant healthcare professionals will be sought on a wider societal, organisational or individual level (patients, their families and healthcare professionals).

Throughout this process, the researchers will refine the developing theory by asking if and in what ways the data support, disprove or shape its development. New CMO configurations may be related, removed, combined, separated or revised.

Analysis of subgroups or subsets

No applicable

Contact details for further information

Jamie Gross

K20040401@kcl.ac.uk

Organisational affiliation of the review

Kings College London

www.kcl.ac.uk/cicelysaunders

Review team members and their organisational affiliations

Dr Jamie Gross. Kings College London

Dr Jonathan Koffman. Kings College London

Type and method of review

Systematic review, Other

Anticipated or actual start date

03 January 2022

Anticipated completion date

02 January 2023

Funding sources/sponsors

Not applicable

Conflicts of interest

Language

English

Country

England

Stage of review

Review Ongoing

Subject index terms status

Subject indexing assigned by CRD

Subject index terms

Acute Disease; Hospitals; Humans; Life Support Care

Date of registration in PROSPERO

16 December 2021

Date of first submission

15 December 2021

Stage of review at time of this submission

The review has not started

Stage	Started	Completed
Preliminary searches	No	No
Piloting of the study selection process	No	No
Formal screening of search results against eligibility criteria	No	No
Data extraction	No	No
Risk of bias (quality) assessment	No	No
Data analysis	No	No

The record owner confirms that the information they have supplied for this submission is accurate and complete and they understand that deliberate provision of inaccurate information or omission of data may be construed as scientific misconduct.

The record owner confirms that they will update the status of the review when it is completed and will add publication details in due course.

Versions

16 December 2021
